# Atlas of Lobular Breast Cancer Models: Challenges and Strategic Directions

**DOI:** 10.3390/cancers13215396

**Published:** 2021-10-27

**Authors:** George Sflomos, Koen Schipper, Thijs Koorman, Amanda Fitzpatrick, Steffi Oesterreich, Adrian V. Lee, Jos Jonkers, Valerie G. Brunton, Matthias Christgen, Clare Isacke, Patrick W. B. Derksen, Cathrin Brisken

**Affiliations:** 1 ISREC—Swiss Institute for Experimental Cancer Research, School of Life Sciences, Ecole Polytechnique Fédérale de Lausanne (EPFL), 1015 Lausanne, Switzerland; 2 The Breast Cancer Now Toby Robins Research Centre, The Institute of Cancer Research, London SW3 6JB, UK; koen.schipper@icr.ac.uk (K.S.); amanda.fitzpatrick@icr.ac.uk (A.F.); clare.isacke@icr.ac.uk (C.I.); 3 Department of Pathology, University Medical Center Utrecht, Heidelberglaan 100, 3584 CX Utrecht, The Netherlands; T.Koorman@umcutrecht.nl (T.K.); P.W.B.Derksen@umcutrecht.nl (P.W.B.D.); 4 Department of Pharmacology and Chemical Biology, University of Pittsburgh, Pittsburgh, PA 15261, USA; oesterreichs@upmc.edu (S.O.); leeav@upmc.edu (A.V.L.); 5 Magee Women’s Cancer Research Institute, Pittsburgh, PA 15213, USA; 6 Cancer Biology Program, Women’s Cancer Research Center, UPMC Hillman Cancer Center, Pittsburgh, PA 15232, USA; 7 Division of Molecular Pathology, The Netherlands Cancer Institute, 1066 CX Amsterdam, The Netherlands; j.jonkers@nki.nl; 8 Oncode Institute, 1066 CX Amsterdam, The Netherlands; 9 Edinburgh Cancer Research UK Centre, Institute of Genetics and Cancer, University of Edinburgh, Crewe Road South, Edinburgh EH4 2XU, UK; v.brunton@ed.ac.uk; 10 Institute of Pathology, Hannover Medical School, Carl-Neuberg-Straße 1, 30625 Hannover, Germany; christgen.matthias@MH-Hannover.de

**Keywords:** invasive lobular breast carcinoma, experimental models, metastasis, PDX, GEMM, tumor organoids, animal models, cell lines, translational research, ELBCC

## Abstract

**Simple Summary:**

Invasive lobular breast cancer (ILC) is the second most common histological subtype of invasive breast cancer, which is noted to have a unique microscopic appearance. The understanding of ILC biology is advancing through the evolution of various experimental models, with the ultimate aim to discover new therapeutic strategies. In this review, we summarize the critical developments in the modeling of ILC. We provide a comprehensive overview of well-established ILC models and discuss different approaches for modeling the disease. We highlight the potential opportunities, the challenges, and the recent advances that have contributed to a better understanding of ILC and envisage the future of ILC modeling.

**Abstract:**

Invasive lobular carcinoma (ILC) accounts for up to 15% of all breast cancer (BC) cases and responds well to endocrine treatment when estrogen receptor α-positive (ER^+^) yet differs in many biological aspects from other ER^+^ BC subtypes. Up to 30% of patients with ILC will develop late-onset metastatic disease up to ten years after initial tumor diagnosis and may experience failure of systemic therapy. Unfortunately, preclinical models to study ILC progression and predict the efficacy of novel therapeutics are scarce. Here, we review the current advances in ILC modeling, including cell lines and organotypic models, genetically engineered mouse models, and patient-derived xenografts. We also underscore four critical challenges that can be addressed using ILC models: drug resistance, lobular tumor microenvironment, tumor dormancy, and metastasis. Finally, we highlight the advantages of shared experimental ILC resources and provide essential considerations from the perspective of the European Lobular Breast Cancer Consortium (ELBCC), which is devoted to better understanding and translating the molecular cues that underpin ILC to clinical diagnosis and intervention. This review will guide investigators who are considering the implementation of ILC models in their research programs.

## 1. Search Strategy and Selection Criteria

References for this review were obtained from Medline, Google Scholar, and SCOPUS searches using the keywords “lobular breast cancer models”, “ILC models”, “genetically engineered mouse model”, “ILC patient-derived xenografts”, “breast cancer xenografts”, “breast cancer organoids”, and “preclinical models” and there was no restriction on year of publication. Databases used included cellosaurus (https://web.expasy.org/cellosaurus/, accessed date: 1 October 2021) [1], German Collection of Microorganisms and Cell Cultures GmbH (DSMZ, https://www.dsmz.de/), American Type Culture Collection (ATCC, https://www.lgcstandards-atcc.org/?geo_country=ch, accessed date: 1 October 2021), Rikagaku Kenkyūjo Cell Bank (RIKEN, https://cell.brc.riken.jp/en/), European Collection of Authenticated Cell Cultures (ECACC, http://www.hpacultures.org.uk/collections/ecacc.jsp, accessed date: 1 October 2021), Japanese Cancer Research Resources Bank (JCRB, https://cellbank.nibiohn.go.jp/english/cellsearch_e/, accessed date: 1 October 2021), Interlab Cell Line Collection (ICLC, http://www.iclc.it/Listanuova.html), Korean Cell Line Bank (KCLB, http://cellbank.snu.ac.kr/english/index.php, accessed date: 1 October 2021), the Mouse Genome Database (MGD, http://www.informatics.jax.org/, accessed date: 1 October 2021), Cell Model Passports (https://cellmodelpassports.sanger.ac.uk/passports/SIDM00241, accessed date: 1 October 2021), the SUM Breast Cancer Cell Line Knowledge Base (SLKBase, https://sumlineknowledgebase.com/, accessed date: 1 October 2021), cBioPortal for Cancer Genomics (https://www.cbioportal.org/), the Human Cancer Models Initiative (HCMI, https://ocg.cancer.gov/programs/HCMI, accessed date: 1 October 2021), the Cancer Dependency Map https://depmap.org/portal/, the International Agency for Research on Cancer (IARC) *TP53* Database (https://p53.iarc.fr/CellLines.aspx, accessed date: 1 October 2021), Cancer Cell Line Encyclopedia (CCLE) (https://portals.broadinstitute.org/ccle, accessed date: 1 October 2021), PDX Finder (https://www.pdxfinder.org/), DepMap project (https://depmap.org/portal/, accessed date: 1 October 2021), and COSMIC cell-line project (https://cancer.sanger.ac.uk/cell_lines, accessed date: 1 October 2021).

## 2. Introduction

Invasive lobular carcinoma (ILC) is the most frequent special histological breast cancer (BC) subtype and accounts for at least 15% of all female BC cases [2,3,4,5]. The vast majority of primary ILC have a low proliferative index, express the estrogen receptor (ER) and the progesterone receptor (PR), and show only rare amplification of HER2. Compared to patients diagnosed with ER^+^/PR^+^/HER2^−^ no special type invasive BC (NST, also known as invasive ductal carcinoma), those with ILC do not show additional benefit of adjuvant chemotherapy [6], tend to have larger tumors on presentation, an increased number of involved lymph nodes, and lower detection through mammographic screening compared to magnetic resonance imaging (MRI) [7]. ILC is also generally characterized by an increased risk of late recurrence [8], which can in part be attributed to the reawakening of quiescent disseminated tumor cells. Histologically, classic ILC typically shows single-cell infiltration and often a targetoid pattern of invasion [9]. Of note, ILC presents with a unique pattern of metastatic spread to bones, ovaries, peritoneum, leptomeninges, GI tract [10,11,12,13,14,15,16], and rarer sites, including the orbital cavity [11]. Spread to the lungs and other visceral organs is less frequently observed than other ER^+^ BCs [17,18,19]. The reasons for this organotropism are not understood but are likely to be governed by intrinsic anchorage-independent tumor characteristics due to the inherent loss of E-cadherin [20] along with different therapies, systemic factors, and microenvironmental cues priming distinct pre-metastatic niches within different organs [21].

At the molecular level, primary ILC has a distinct genomic landscape from NST [22,23,24]. First, the mutational inactivation of E-cadherin (encoded by *CDH1*) provides a biochemical signature that promotes ILC dissemination through anoikis resistance [25] and amplifies endogenous growth factor receptor (GFR) signals [26,27]. Second, mutations in *ERBB2*, *ERRB3* and/or *PIK3CA* further enhance these oncogenic GFR signals by directly activating the PI3K/AKT pathway. Finally, primary ILCs have distinct immune microenvironments with low levels of tumor-infiltrating lymphocyte (TILs) and a different immune cell composition compared to ER^+^/HER2^−^ NSTs [28,29]. Less is known about the mutational repertoire of ILC metastases. In a recent study, *TP53* and *ESR1* mutations were more frequent in ILC metastases, tumor mutational burden was higher in ILC metastases, and *RHOA* mutations were more frequent in ovarian ILC metastasis [14]. Despite these differences, patients with ILC are treated similarly to patients with NST, which highlights the need to understand the molecular underpinnings of ILCs to inform advanced treatment options.

**Figure 1:**
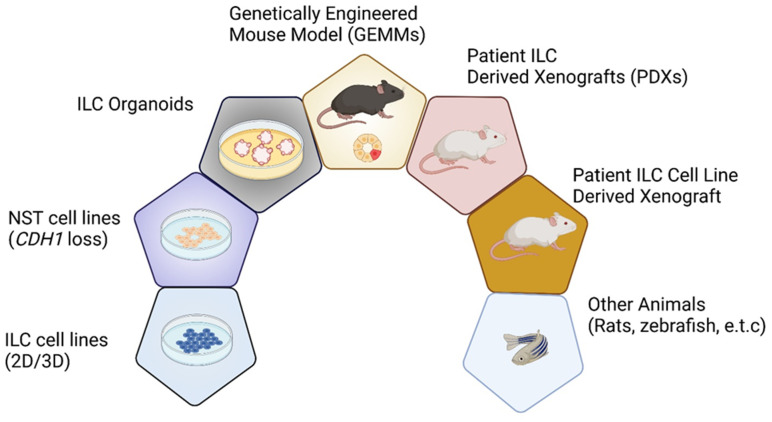
Overview of ILC model systems. Schematic representation of seven key experimental models used for ILC research. Abbreviations: NST, no special type; GEMMs, genetically engineered mouse models; ILC, invasive lobular carcinoma; PDXs, patient-derived xenografts; 2D, two-dimensional; 3D, three-dimensional.

Laboratory models are vital for helping us understand the biology of ILC (Figure 1) [2]. Despite attempting to develop ILC cell lines using many different culture conditions and varying starting material, the generation of long-lived established ILC cell lines has been unsuccessful. Moreover, the number of relevant and well-established ILC models remains limited due to the lower incidence of ILC, fewer ILC patients in clinical trials, and the intrinsic characteristics of ILCs that do not readily transplant/grow as organoids. This study systematically reviews the in vitro, ex vivo, and in vivo experimental models, aiming to provide a comprehensive summary of the existing ILC models, exploring their strengths and weakness, and paying particular attention to the recent and future advances of ILC modeling (Figure 1 and Table 1).

## 3. In Vitro Cell-Based ILC Models

Human-derived cell lines have been the mainstay of cancer research [30]. However, the list of authenticated ILC lines is short, with limited patient-related clinical and treatment information available. Only a handful of ILC cell lines have been extensively studied. ILC cell lines proliferate significantly slower than their NST line counterparts, and only a few of them derived from primary tumors but most from pleural effusions or ascites, i.e., late metastatic disease (Table 2). In addition, almost all of them were derived from Caucasian females [31]. Although 90% of ILCs are ER^+^/PR^+^, only a minority of the available models express ER. As expected, most of them have pathogenic *CDH1* mutations, but although only 8% of clinical ILCs have altered *TP53* [23,32], the widely used ILC models express mutant *TP53* (Table 2, Supplementary File S1).

### 3.1. E-cadherin-Deficient Well-Characterized ILC Cell Lines

SUM-44PE are widely used ILC cells isolated from a pleural effusion of a patient unresponsive to both endocrine treatment and chemotherapy with ER^+^ ILC in 1993. SUM-44PE cells possess a *CDH1* point mutation, leading to no detectable E-cadherin protein levels. Moreover, these cells harbor truncating *TP53* mutation, which leads to low transcripts and p53 expression [48]. SUM-44PE expresses high ER protein levels, and both *CCND1* (Cyclin D1) and *FGFR1* are amplified [49]. Interestingly, a deleterious *ESR1^Y537S^* mutation, frequently found in refractory ILC [50], pre-exists in a fraction (one out of a million) of in vitro 2D-cultured SUM-44PE cells and is readily detectable within 12 weeks following the transfer of the cells to estrogen-free medium [51]. Copy number changes, mutational data, and genome-wide expression have been extensively described at the SUM Breast Cancer Cell Line Knowledge Base (SLKBase) [52].

**Figure 2:**
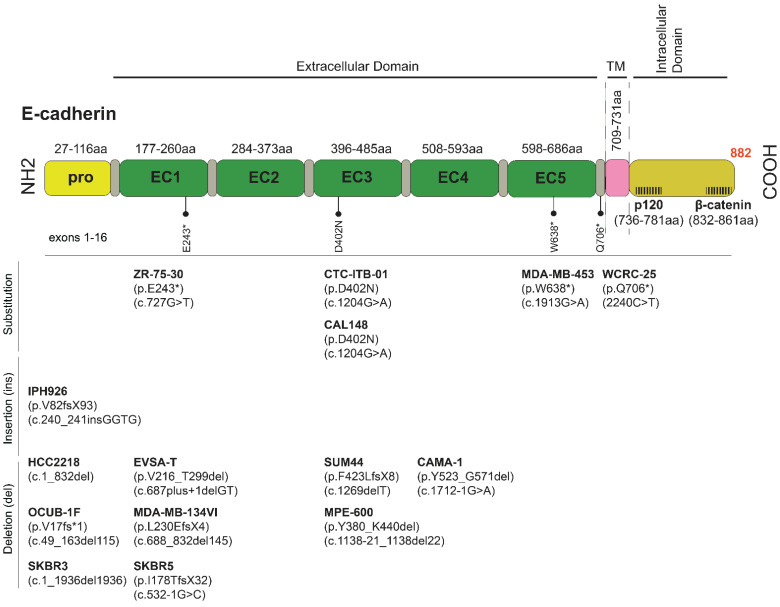
*CDH1* mutations and sequence variants commonly found in ILC and ILC-like cell lines. Abbreviations: fs, frameshift; EC, extracellular domain; TM, transmembrane; *, termination, stop codon; X, termination; p, protein reference sequence; c, coding DNA reference sequence; del, deletion.

IPH-926 cells, derived from the ascites of a patient with an endocrine and chemotherapy-resistant metastatic ILC relapse, are the second well-characterized ILC model [34]. IPH-926 cells are triple negative (TN), like the therapy-refractory ILC relapse of the corresponding patient. However, the corresponding primary tumor of the patient, which was diagnosed 16 years before the establishment of the IPH-926 cell line, was a grade 1 ER^+^ ILC [34]. Accordingly, IPH-926 cells reflect the tumor biology of a late-stage ILC after many years of tumor progression and ER status conversion under clinical therapy. IPH-926 cells harbor a *CDH1* frameshift mutation and lack E-cadherin protein expression (Figure 2). The same *CDH1* frameshift mutation was demonstrated in the primary ILC of the corresponding patient [34]. IPH-926 cells display a luminal gene expression profile, despite the loss of ER and lack of PR and AR expression [53]. IPH-926 cells lack *PIK3CA* mutation, but the PI3K/AKT pathway is intrinsically activated [27]. Notably, IPH-926 are temperature-sensitive due to endogenous expression of a temperature-sensitive (ts) p53 mutant (ts *TP53* E285K), which can toggle between wild-type function and loss of function states, depending on the cell culture temperature [54]. Interestingly, this *TP53* mutation occurred as a secondary genetic event during the clonal evolution of the respective ILC in the corresponding patient. The occurrence of the *TP53* E285K mutation was associated with a morphological shift to a G3-differentiated ILC with pleomorphic features in the corresponding clinical tumor specimens. Furthermore, IPH-926 cells display overexpression of the BC anti-estrogen resistance 4 (BCAR4) gene, which has been linked to hormone-independent tumor cell growth [55,56,57], display overexpression of the MDR1/ABCB1 drug transporter, and actively extrude conventional chemotherapeutic agents, such as doxorubicin, in an MDR1/ABCB1-dependent manner [56]. Overexpression of MDR1/ABCB1 in IPH-926 is likely related to palliative poly-chemotherapy administered to the corresponding patient before this cell line was derived from ascites. Finally, IPH-926 cells also lack beta-catenin protein expression and are tumorigenic in SCID female mice [34].

The MDA-MB-134-VI line is a well-accepted ER^+^ ILC model, yet derived from the pleural fluid of a patient diagnosed with a primary ductal papillary mammary carcinoma. Molecular reclassification revealed that these cells belong to the luminal molecular subtype, and based on genetic and expression profiles, resemble classic lobular carcinomas (CLC) [58]. Concomitantly, MDA-MB-134-VI does not express E-cadherin due to truncating mutations (deletion of exon 6) and appears to be de novo tamoxifen-resistant [33,59]. In 2D culture, these cells form grape-like clusters, and E-cadherin/p120 catenin dual staining shows diffuse cytoplasmic p120, further confirming that MDA-MB-134-VI represents an ILC cell line [60]. MDA-MB-134-VI cells also show *FGFR1* amplification and have been proposed as a model system for the preclinical investigations of BCs with FGFR1 overexpression [58]. However, these cells also contain an oncogenic *KRAS* mutation uncommon in ILC and may override the proximal GFR dependence and induce attenuation of drugs targeting FGFR1 or PI3K/AKT [61]. Additional E-cadherin-deficient ILC lines are, to a high degree, not well characterized and not widely used, including UACC-3133, MA-11, EFM-63, and the discontinued HCC-2185 cells (Table 2 and Supplementary File S1).

### 3.2. ILC-like Cell Lines Derived from Clinical NSTs with ILC Molecular Features (Genetic and Expression Profile)

Eleven BC cell lines are annotated as “carcinoma”, “adenocarcinoma” or “ductal carcinoma”, with no detailed pathology report or molecular analysis of the corresponding clinical tumor specimens available, yet recent data show that they have key ILC genetic features, such as *CDH1* mutation.

BCK4 cells derived from the solid tumor xenograft of a poorly differentiated mucinous adenocarcinoma readily switch to an ILC morphology with mucinous features when mice are supplemented with E2 [38,62]. BCK4 cells exhibit a partial tamoxifen agonism similar to MDA-MB-134-VI cells, express low levels of PR, its growth is markedly accelerated by E2, and have been suggested as a *TP53* wild type ILC model [63].

SK-BR-3 is an ER^−^ cell line with an amplified ERBB2 locus that overexpresses Her-2/neu and is sensitive to lapatinib [64]. SK-BR-3 cells have been demonstrated as a valuable preclinical model to explore resistance mechanisms to HER2-targeted therapies and screen for new drugs [35,65]. SK-BR-3 cells do not express E-cadherin due to a large homozygous deletion commonly found in ILCs and therefore form loosely cohesive grape-like or stellate structures [35,66,67]. They express well-differentiated luminal epithelial-like phenotype markers but do not grow well in vivo [68], with few exceptions, including the formation of poorly differentiated tumors in immunocompromised mice [69].

MDA-MB-453 cells were obtained from the malignant pericardial fluid consistent with the metastatic propensity of ILCs to the serosal cavities. As this cell line expresses androgen receptor (AR), it has been traditionally used as an apocrine BC model [70]. Notably, apocrine differentiation has also been described in the pleomorphic ILC [71]. Due to nonsense *CDH1* mutation, the cells present with non-functional E-cadherin [72]. They belong to the grape-like class of cell lines, which form colonies with poor cell–cell contacts [73]. Notably, cells with grape-like phenotype typically express moderate levels of HER2 [73]; herein, MDA-MB-453 express both HER2 and HER3 proteins [74,75]. Interestingly, MDA-MB-453 cells also have an inactivating *PTEN* mutation found in at least 10% of ILCs [22].

ZR-75-30 is a luminal-like cell line derived from the ascitic fluid of a woman with primary NST [43]. It is of particular interest as it expresses ER and shows a rounded epithelial morphology [43,76]. ZR-75-30 cells also overexpress HER2, which has also been found amplified in ILC [49,69] and have traditionally been used as an ER^+^ BC model insensitive to tamoxifen, yet its growth is stimulated by progesterone [77].

CAMA-1 was derived from a solid BC with scirrhous regions. CAMA-1 cells are ER^+^/PR^+^, responsive to estrogen, and sensitive to growth inhibition by tamoxifen. Moreover, *CDH1* is genetically inactivated via biallelic in-frame mutations and deletion of exon 6, resulting in a shortened, non-functional E-cadherin protein yet detectable at the cell membrane and weakly diffuse in the cytoplasm [78]. Morphologically the cells grow as small, loosely packed epithelial-like cells [79] with wild-type *GATA3* consistent with fewer *GATA3* mutations found in primary ILC versus NST tumors [22] and are HER2-negative.

600MPE is an ER^+^ line with a large *CDH1* deletion, resulting in a shortened, non-functional E-cadherin protein, yet the original clinical diagnosis was NST BC [45].

The ER^+^ circulating tumor cell line CTC-ITB-01 was derived from the peripheral blood of a female patient with bilateral mammary carcinoma of a well-differentiated ILC on the left breast and NST on the right breast [80]. CTC-ITB-01 carries a “private” variant in the *CDH1* gene (c.1204G>A; p.D402N), an uncharacterized missense pathogenic (predicted) mutation in the E-cadherin extracellular domain 3 (EC3) identical to the one found in CAL-148 cells. Notably, the EC3 domain is necessary for homophilic E-cadherin adhesion, and it remains to be seen how this model will represent ILC phenotypes. Additionally, ILC-like cell lines, with genetic *CDH1* inactivation and a rounded epithelial-like morphology that warrant further molecular characterization, include EVSA-T [41], OCUB-1F [47], CAL-148 [42], and HCC2218 [44] (Table 2).

Together, *CDH1* defective ILC-like cell lines are often TN, androgen receptor-positive (AR^+^), or overexpress HER2. Interestingly, clinical studies have shown that primary TN-ILC, at the molecular level, is characterized by increased AR signaling and frequent alterations in HER2 network proteins [81]. In addition, *HER2/3* mutations are relatively common, particularly in the pleomorphic ILC subtype [3,82,83,84,85,86], and HER2 overexpression is reported in primary ILCs [62].

### 3.3. Epigenetic Inactivation of E-Cadherin

Along with truncating mutations and allelic *CDH1* loss, epigenetic silencing is an additional but less common and questionable mechanism for the lack of E-cadherin expression [22]. Methylation of 5′ CpG islands has been described for many carcinomas, including gastric [87], skin [88], and ILC [89]. Epigenetic silencing of E-cadherin is considered a non-driver and late event in ILC progression [20]. This epigenetics phenomena has been found in six ILC-like cell lines. These BC cell lines with *CDH1* promoter methylation and without detectable E-cadherin protein were all derived from tumors with a non-ILC primary diagnosis (BT549 [46], SUM159PT [90], CAL-120 [91], MDA-MB-157 [92,93], MDA-MB-436 [39], and HS578T [94]). These lines would need further evaluation as putative ILC models (see Table 2 and Supplementary File S1).

### 3.4. ILC Lines with Proficient E-Cadherin and Defective Adherens Junctions

Impaired adherens junctions, a hallmark of ILC phenotype, may also be affected by α-catenin (*CTNNA1*) loss rather than lack of E-cadherin itself. For example, aberrant E-cadherin-dependent cell–cell adhesions in the PC-3 prostate cancer line are dictated not by loss of E-cadherin but by the lack of α-catenin [95]. Likewise, there is one ILC line and three ILC-like E-cadherin positive BC lines with non-functional adherens junctions. E-cadherin expressing MDA-MB-330 cells were derived from pleural effusion of a patient with ILC and grew out as polygonal cells in islands [39]. However, they have non-functional adherens junctions because of a biallelic *CTNNA1* mutation (truncating *α-catenin*) to impair E-cadherin function [2] and have been used as an ILC model in 2D and 3D in vitro studies [63]. In these cells, *ERBB2* is amplified, and β-catenin is expressed in the absence of α-catenin. MDA-MB-468 is an Rb-deficient cell line that expresses E-cadherin and has acquired *CTNNA1* bi-allelic mutations [39]. In vitro, MDA-MB-468 cells show a typical ILC-type growth. However, in vivo, they only grow if they are co-injected with 3T3HAS3 fibroblasts [96]. This line was initially classified as basal-like, but recent reclassification based on the whole transcriptome profile showed more similarity to the luminal/HER2 group [97]. Two additional less characterized ILC-like cell lines with *CTNNA1* mutant are MDA-MB-157 [92] and HCC1187 [44].

### 3.5. ILC Cell Lines Developed from Patient-Derived Xenografts (PDXs)

Stable cell lines can be generated once they have passed via in vivo xenograft growth. However, with regard to ILC, only one cell line (UCD178) has been generated directly after fourteen months of continuous in vitro culturing of dissociated PDX xenografted cells [98].

### 3.6. Experimental CDH1 Downregulation/Deletion in E-Cadherin-Positive Non-ILC Cells

Loss of E-cadherin expression is a hallmark of ILCs. Therefore, silencing or knockout of *CDH1* in non-ILC cells has been considered a reasonable alternative to model ILC mechanistically. E-cadherin-null isogenic partners have been generated for human ER^+^ NST lines MCF7 [90], T47D, and MDA-MB-468, the murine 4T1 cells [99], as well from the non-malignant breast epithelial MCF10A cells and primary cells isolated from normal breast tissue [100,101,102,103,104,105]. Interestingly, E-cadherin deficiency in non-ILC cells is insufficient to induce an EMT [100]. For example, the E-cadherin deficiency MCF7 cells display rounded morphology, as seen in ILC cells harboring naturally occurring E-cadherin mutations [90], and grow 3.5-fold less than the controls when grafted in the mouse milk ducts [106]. Moreover, lentiviral knockdown of E-cadherin expression in NST TN PDX organoids significantly increases invasion and dissemination and decreases colony formation [107].

Notably, isogenic MCF7 cells with frameshift *CDH1* mutations and concomitant loss of E-cadherin expression (Supplementary File S1) have been proven valuable tools for biochemical and synthetic lethality studies [90,108]. Notably, both ROLO (NCT03620643) and ROSALINE (NCT04551495) clinical trials targeting ROS1 are based on synthetic lethality studies [27,90,109].

### 3.7. Ongoing Efforts for the Generation of Additional ILC Lines

The generation of additional BC cell lines from primary and metastatic tumors is essential for experimental ILC studies but has proven challenging. However, a recent exception is the generation of WCRC25, an ILC cell line generated from a pleural effusion from a patient with ILC (Elangovan et al., submitted abstract for SABCS 2021). The cell line is characterized by loss of E-cadherin due to a missense mutation in *CDH1* coupled with the loss of heterozygosity of the other allele (Table 2). Like the majority of BC cell lines, WCRC25 has a *TP53* mutation. Although the pleural effusion was weakly ER^+^, WCRC25 lacks ER expression and does not respond to estrogen. RNA sequencing revealed activation of AKT signaling, which should be further explored in future studies.

## 4. ILC Cell Line-Based 3D Models and Ex Vivo Patient-Derived 3D Tumor Organoids (PDOs)

Recently, multidisciplinary efforts to optimize experimental models have led to the emergence of novel approaches involving 3D culture formats, organoids, spheroids, organotypic cultures of tissue slices or co-cultures, and bioengineered materials which more faithfully reflect the intra-tumoral heterogeneity and the spatial, biochemical, and mechanical properties of the malignant tumor than 2D plastic dish cultures [110,111,112,113,114,115]. Three-dimensional culturing of ILC cell lines has sparsely been reported; literature is currently limited to the comprehensive 2D and 3D phenotypic characterization of only four ER^+^ human ILC cell lines: MDA-MB-134-VI, SUM-44PE, MDA-MB-330, and BCK4 (Table 3, Supplementary File S1) [63]. The growth of NST and ILC cell lines in 3D suspension culture environments confirmed the remarkable anchorage-independent growth characteristics unique to ILC cells [63]. Although ILC cell lines show different morphologies when they grow in 3D extracellular matrix (ECM) gels and divergent adhesive properties on matrix proteins, overall display a “grape-like” morphology previously described for cells with poor cell–cell adhesion [63]. Moreover, ILC lines show limited migration in the commonly used wound-scratch assay compared to NST cells. Similarly, in mammosphere assays, E-cadherin expressing cells successfully propagate as long-term mammosphere cultures. In contrast, ILC-like SKBR3 and MDA-MB-468 cells form cell clumps that can be disaggregate mechanically, and only re-expression of E-cadherin allows them to form mammospheres [116]. The 3D ECM growth of other human ILC cell lines has not been systematically analyzed.

Primary patient-derived BC organoid (PDO) models better conserve the original genetic status of the primary tumor, probably because the PDO models are cultured relatively short-term and in the presence of a serum-free medium. Recently, a seminal study developed a living biobank of breast cancer PDOs [117]. In this resource, 26 primary ILCs were reported, of which 18 were established as PDO ILC models and were found to have a unique growth pattern, being grape-like compared to the dense clusters formed by NST organoids (Table 3, Supplementary File S1). Similarly, another recent report on ILC PDO reveals that this will be a growing resource for research [118]. Lee/Oesterreich laboratories have developed PDOs from over 20 cases of ILC and find similar grape-like growth patterns and DNA mutations like those in human ILC (unpublished). Notably, culturing in 3D laminin-rich matrices, PDO models better retain the histological organization, differentiation status, and morphologic heterogeneity observed in primary tumors [119]. Although short-term propagation of various freshly dissected primary carcinomas has been successful, propagation for an extended period has remained challenging.

With PDO technology, primary tumors are typically cultured in a serum-free medium supplemented with growth factors and basement membrane extract (BME or Matrigel) matrices. As alternatives to Matrigel, several groups have been experimenting with the use of neutral hydrogels such as polyethylene glycol (PEG) [120], polyisocyanopeptide (PIC) hydrogels [121], and sodium alginate [122]. In a recent study, samples from 63 breast cancer patients, including ten ILCs embedded in alginate, continuously grew in culture for one month [123]. The architectural features of the encapsulated tissue microstructures were similar to the original patient tumors, and the organoids were responsive to endocrine treatments demonstrating active ER signaling.

Finally, collaborative efforts are needed to create and document additional ILC organoids; the National Cancer Institute, for example, is generating repositories of organoids from primary and metastatic BC tissues and blood specimens see Appendix A (https://pdmr.cancer.gov). More recently, within the ILC research community and the LOBSTERPOT initiative (https://www.cost.eu/actions/CA19138/), additional (unpublished) ILC PDO models have become available. They contain primary and metastatic ILC PDOs that can also be used as PDX models (Supplementary File S1).

## 5. ILC Mouse Models

### 5.1. Genetically Engineered ILC Mouse Models

**Figure 3:**
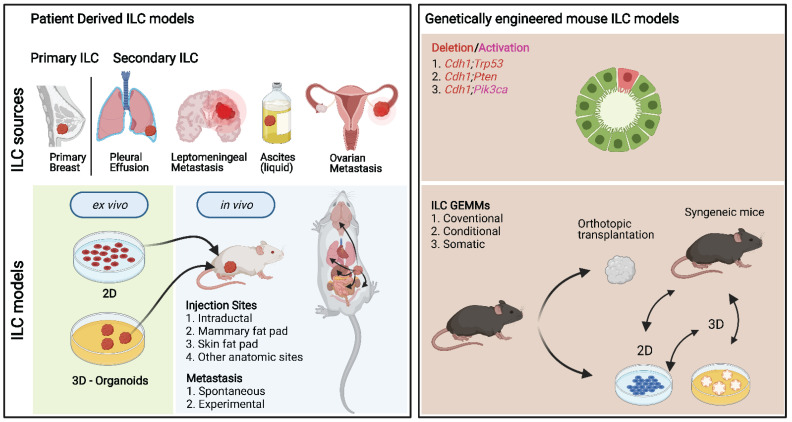
Schematic overview of different key ILC models. Left: Patient-derived ILC models. Primary and secondary ILC tissues are commonly used to establish ILC lines, organoids, xenografts, and PDXs. Right: Genetically engineered mouse ILC models (GEMMs). To study the direct consequence of genetic inactivation of E-cadherin, encoded by the *Cdh1* gene, and other frequently mutated genes in invasive lobular carcinoma (ILC) of the breast, various GEMMs have been developed.

Over the last two decades, substantial progress has been made in generating genetically engineered mouse models (GEMMs) of ILC (Figure 3). The prevalence of E-cadherin loss in ILCs made it a logical target for genetic manipulation; however, initial mouse models showed that somatic Cre recombinase-mediated loss of E-cadherin in the mammary epithelium is insufficient to induce tumor formation [25,124]. Moreover, somatic inactivation of E-cadherin in the context of the MMTV-PyMT did not lead to the development of ILC (Dr. R. Kemler, personal communication). However, stochastic loss of E-cadherin in combination with deletion of the tumor suppressor p53 resulted in the first GEMM of ILC, which showed a substantial acceleration of tumor development compared to the loss of p53 alone [25]. In this GEMM, the cytokeratin 14 (K14) promoter sequence was used to drive the expression of Cre recombinase, which meant that mice not only developed mammary tumors but also skin tumors (Table 4, Supplementary File S1). 

Importantly, the ILC lesions developed in these mice induced metastatic dissemination to the full spectrum of the human metastatic ILC condition. In the next iteration, the use of the mammary gland-specific whey acidic protein (WAP) promoter sequence to drive Cre expression gave rise exclusively to mammary tumor formation [131]. The mammary tumors induced by combined loss of E-cadherin and p53 display a mix of morphologies, with ER-negative pleomorphic ILC being the most common. Despite being a main driver event, loss of E-cadherin expression is not seen in all ILCs. Some ILCs retain membranous E-cadherin expression, while they do display the typical non-cohesive lobular growth pattern. Apart from extracellular mutations in *CDH1* leading to disruption of homotypic interactions or mutations leading to truncations of the intracellular E-cadherin domains, disruption of other members of the adherens junction might underlie the lobular growth pattern in these E-cadherin-proficient ILCs. However, when inactivated in conjunction with p53, loss of p120 did not result in tumors with an ILC-like morphology [132].

Moreover, deletion of p120 catenin in addition to E-cadherin and p53 did not accelerate tumor formation, and the morphology of these triple-knockout tumors was predominantly sarcomatoid [133]. This indicated that cytoplasmic p120 catenin is required for the development of ILC in mice. A more promising candidate appears to be α-catenin, an important link between the adherens junction and the actin cytoskeleton. Loss of α-catenin in p53-deficient mammary tumor cells resulted in anchorage independence, constitutive actomyosin contraction, and an ILC-like morphology [134].

Human ILCs are commonly associated with PI3K pathway mutations [3,22,24], making these attractive targets to model ILC in mice. Multiple mouse models of ILC have been generated by combining mammary-specific loss of E-cadherin with either expression of hotspot mutations of *Pik3ca* (E545K or H1047R), *Akt* (E17K), or somatic deletion of the tumor suppressor *Pten* [125,126,127]. In all models, the combination of E-cadherin loss and PI3K pathway activation decreased mammary tumor latency and promoted the development of tumors with classic ILC features. Tumors induced by E-cadherin loss and expression of *Akt* (E17K) have a more solid ILC morphology and typically higher proliferation rates than tumors induced by expression of mutant *Pik3ca* [126] or loss of *Pten*. In addition, tumors induced by E-cadherin loss and expression of mutant *Pik3ca* or loss of *Pten* are ER^+^ and have a gene expression profile similar to the luminal A subtype. In addition, the mutant *Pik3ca* model has an immune-related molecular subtype combined with activation of immune checkpoint pathways, making it an interesting model to study the therapeutic potential of immune checkpoint inhibitors in ILC [126]. These tumors have not been tested for responsiveness to hormonal therapies, so it remains to be determined whether they can be used to study endocrine therapy resistance mechanisms. Interestingly, mutant *PIK3CA* has been proven as a model for studying the immune-related molecular ILC subtype [126].

To identify novel ILC driver genes, an in vivo insertional mutagenesis screen yielded multiple candidates, several of which were subsequently used to generate novel GEMMs of ILC [128]. Two of these models, which combined mammary-specific loss of E-cadherin with overexpression of truncated variants of *Mypt1* or *Aspp2*, showed that a reduction in actomyosin contractility is sufficient to drive the malignant transformation of E-cadherin deficient mammary epithelial cells [128,129]. The tumors typically displayed classic ILC features but were not tested for ER expression or dependency. Interestingly, both models showed rapid tumor initiation but slow tumor growth, a common characteristic of ILCs. Additional loss of PTEN resulted in faster tumor initiation and larger tumors [129]. In vivo validation of a third hit from the insertional mutagenesis screen, TRPS1, showed that while TRPS1 expression is essential for the survival of mammary epithelial cells, combined loss of TRPS1 and E-cadherin expression resulted in accelerated tumor development [130].

#### 5.1.1. Orthotopic Transplantation of Mouse ILC Tumor Fragments into Syngeneic Mice

One of the main limitations of traditional GEMMs is developing mammary tumors in multiple glands. These tumors arise and progress at different rates complicating both the ability to test for drug efficacy and studying cancer metastasis. Transplantation of tumor fragments from mouse ILCs into syngeneic wild-type mice has therefore been utilized to study mechanisms of acquired drug resistance [90,135,136] and metastasis [137] (Figure 3). One particular strength of this method is that these processes can be carried out in an immunocompetent context enabling interrogation of novel immunotherapies and the critical role of the immune system in cancer metastasis [29,138,139,140]. The transplantation of tumor fragments works robustly for the p53-deficient mouse ILC models but is challenging with models that have a classical ILC morphology. It is currently unclear why the classic ILC models do not grow out consistently.

#### 5.1.2. GEMM-Derived ILC Lines

In addition to in vivo studies, ILCs from the GEMMs mentioned above have also been used to derive 2D and 3D cell lines [25,125,126,131]. The ILC cell lines derived from K14-Cre;Cdh1^F/F^;Trp53^F/F^ (KEP) tumors have been used extensively in follow-up studies as they can be readily genetically modified and transplanted into mice, making them ideal for mechanistic studies [108,132,141,142]. The classic ILCs from WAP-Cre;Cdh1^F/F^;Pten^F/F^ and WAP-Cre;Cdh1^F/F^;R26-LSL-Pik3ca^H1047R^ mice have been successfully used to culture organoids/spheroids [125,126], but they do not give rise to classic ILCs when transplanted back into mice, suggesting their limited use for in vivo studies (Table 4 and Supplementary File S1).

#### 5.1.3. Somatic ILC Models

GEMMs have played a critical role in advancing our understanding of tumorigenesis, but they have several limitations. Traditional GEMMs are notoriously time-consuming and expensive to establish. While the development of new strategies such as in vitro modification of embryonic stem cells derived from existing GEMMs has accelerated the process, it remains costly and laborious [143]. It has long been known that the mammary gland structure allows for the administration of exogenous substances via intraductal injections [144]. However, it was not until recently that this administration route was used to generate somatic mouse models of ILC [127]. Intraductal injection of viruses encoding for Cre-recombinase enables control of the number of mammary glands that will develop tumors, thus overcoming a limitation of traditional GEMMs.

Furthermore, the injection of lentiviruses encoding candidate oncogenes allows for rapid validation of driver genes or mutated variants without having to engineer new GEMMs [145]. Finally, the development of CRISPR technology has exponentially increased the possibilities for somatic modification of genes and their expression. Intraductal injection of lentiviruses encoding guide RNAs targeting *Pten* or *Myh9* in mice with mammary-specific loss of E-cadherin and expression of Cas9 resulted in the formation of tumors with a classic ILC morphology [127,128]. The limitation of this technique is that the efficiency is lower than the traditional GEMMs, as not all injected glands develop tumors. More recent advances in genome engineering, such as CRISPR-Cas9-based editors [146,147], have not yet been used to generate ILC mouse models, but it has been shown that they can be used to generate *PIK3CA* hotspot mutations frequently seen in ILC patients [146].

## 6. Modeling ILC with Xenografts

Xenograft models in which cancer cells typically grow in immunodeficient mice have revealed important aspects of ILC progression and therapy resistance mechanisms. Four conventional routes of inoculation have been widely used to generate xenografts by surgery: systemic (intracardiac injections through the left ventricle and intracarotid through the internal carotid artery), local (renal capsule, intracranial, intra-bone marrow, and intrailiac artery injection), under the skin (intradermic or subcutaneous), orthotopic (mammary fat pad) and inside the milk ducts (Figure 3). Specifically for ovarian hormone-dependent ER^+^ BCs, often, recipient mice require exogenous E2 supplementation for successful tumor engraftment and growth [31]. These approaches mainly mimic E2 levels found in premenopausal women in the luteal phase and frequently have deleterious effects on the host. Researchers often use medium rich in selected growth factors, basement membrane extract (BME), and Matrigel to increase engraftment efficiency. However, not well-defined growth factors found in murine-derived BME and batch-to-batch variability might support the preferential engraftment of specific ILC cell types and might lead to a lack of reproducibility.

### 6.1. Cell Line-Derived Xenografts

Due to low tumor take rates, only a few ILC xenografts have been established and are currently of limited utility for ILC research [31,148]. Among the few established xenografts, following the conventional subcutaneous or fat pad implantation approaches, are the IPH-926 cells [54]. IPH-926 cells injected subcutaneously recapitulated the linear cord invasion pattern and the occasional intracytoplasmic lumina with central mucoid inclusions, characteristic of human ILCs [34,54]. Upon E2 supplementation, the BCK4 cells injected into the mammary fat pad of NOD-SCID mice form ILC with mucinous features [38,149], whereas CAMA1 cells grow poorly as xenografts [150]. MDA-MB-453 cells, injected subcutaneously, can only grow if suspended into Matrigel [151] while growing without hormone supplementation when injected into the mammary fat pads of cycling female nu/nu mice [152].

Recently, it was demonstrated that the microenvironment is a key determinant for the growth of ER^+^ BC by injecting normal breast cells or BC cells directly into the primary milk ducts, which resemble the specific anatomic site where BCs arise from [153,154,155,156,157]. It has been shown that intraductal injection enables the physiological growth of ER^+^ BC cells and facilitates the study of the natural BC progression from primary tumor formation to dormancy and metastases in clinically relevant organs [106,148,156,158,159]. Hitherto a panel of BC cell lines representative of different subtypes have been injected directly into the milk ducts of NSG female mice and led to their robust in vivo growth without E2 repletion, including two widely used ILC cell lines (SUM-44PE and MDA-MB-134-VI) and one ILC-like (MDA-MB-453) [154,160]. Histological analysis revealed that intraductal xenografts progress from in situ to the invasive phase over several weeks and faithfully resemble human lobular characteristic morphological patterns of a single file, pagetoid, and targetoid spread [160,161]. Morphologically, ILC cell lines colonize the ductal tips and give rise to grape-like structures, whereas E-cadherin-proficient NST cells cause widespread dilation of host murine ducts. Notably, ILC xenografts stained highly positive for ER and showed E2-inducible PR expression confirming the estrogen responsiveness of this model. SUM-44PE and MDA-MB-134-VI metastasized to clinically relevant organs, including the leptomeninges, GI tract, and ovaries. Over 10–12 months after initial intraductal injection, tumor burden in distant organs was increased to levels comparable to those seen in the primary tumor. Using the intraductal approach, global gene expression profiling of pure primary ILC and NST xenografted cancer cells revealed ECM remodeling as a key tumor cell-intrinsic ILC feature. ILCs secrete proteins and enzymes that control their own matrix, which opens new therapeutic strategies [160].

### 6.2. Patient-Derived Xenografts (PDXs)

Patient-derived xenograft (PDX) models overcome the limitations of cell line-derived xenografts with essential applications to preclinical studies and inform patient care [162,163,164]. Typically, freshly dissected primary or metastatic BC tissue is implanted directly into an animal, typically an immunocompromised mouse. Notably, the generated PDXs retain the genetics, the polyclonality, and the intratumor heterogeneity of the originating human tumors [165,166,167,168]. PDXs hold promise as a discovery and validation platform across multiple institutions [148,169,170]. Biologically, the most critical challenge in developing ILC PDXs is the substantially lower engraftment rate of ER^+^ compared to TN or HER2^+^ BCs. Moreover, ILCs develop over several years, so not surprisingly, ILC xenografted tumors are notoriously slow-growing. To date, only a few ILC PDXs have been described (Table 5).

For testing new agents and drug responses, BC fragments from a cohort of 200 samples with a range of stages and histologies, including three TN-ILCs implanted directly into the interscapular fat pad of adult female Swiss nude mice [171,172,173]. The generated PDXs recapitulated the features of the original tumors, and the HBCx-7 model (p53 wild-type) responded well to docetaxel treatment, pointing to a valuable preclinical drug testing tool. ILC PDXs have also been generated subcutaneously in severe immunocompromised NSG female mice in the presence of 8 mg/mL of E2 in the drinking water [174]. Several PDXs, including seven ILCs, were also generated by initially embedded tumor samples in Matrigel and then implanted subcutaneously into female NSG mice (Table 5) [175].

**Table 5:** ILC Cell Line-Derived and Patient-Derived Xenografts. Abbreviations: ΜA, malignant ascites; OvM, ovarian metastases (*murine); BrM, brain metastases; ER, estrogen receptor; PR; progesterone receptor; ChR, chest recurrence; NST, non-special type; SkR, Skin recurrence (chest wall); PrBC, primary breast cancer; PE, pleural effusion; LCIS; lobular carcinoma in situ; n/a, not available; un: unspecified; SkC, skin right clavicle; AF, Ascitic fluid; EPFL, The École polytechnique fédérale de Lausanne; ISREC, Swiss Institute for Experimental Cancer Research; IC, Institute Curie; MHH, Institute of Pathology, Hannover Medical School; MBRC, The NIHR Manchester Biomedical Research Centre; WUSTL, Washington University in St. Louis; HCI, Huntsman Cancer Institute; BCM, Baylor College of Medicine; ns, not specified; Ref, reference(s). See also Supplementary File S1.

Name	Tumor	Tissue	Biomarker (Model)	Laboratory/Institute	Implantation Site	Ref.
**Xenografts (cell lines)**						
SUM-44 PE	ILC	PE	ER^+^/PR^+/−^/HER2^−^	Prof. C. Brisken/EPFL-ISREC	Milk ducts	[160]
MDA-MB-134-VI	ILC	PE	ER^+^/PR^+/−^/HER2^−^	Prof. C. Brisken/EPFL-ISREC	Milk ducts	[160]
IPH-926	ILC	MA	ER^−^/PR^−^/HER2^−^	Prof. M. Christgen/MHH	Subcutaneous	[34]
**PDXs**						
T69	ILC	PrBC	ER^+^/PR^+^/HER2^−^	Prof. C. Brisken/EPFL-ISREC	Milk ducts	[176]
T73	ILC	PrBC	ER^+^/PR^−^/HER2+	Prof. C. Brisken/EPFL-ISREC	Milk ducts	[176]
T78	ILC	PrBC	ER^+^/PR^+^/HER2^−^	Prof. C. Brisken/EPFL-ISREC	Milk ducts	[176]
T85	ILC	PrBC	ER^+^/PR^−^/HER2^−^	Prof. C. Brisken/EPFL-ISREC	Milk ducts	[176]
T86	ILC	PrBC	ER^+^/PR^+^/HER2^−^	Prof. C. Brisken/EPFL-ISREC	Milk ducts	[176]
LA-PDX1	ILC	PrBC	ER^+^/PR^+^/HER2^−^	Prof. R. Iggo/BCI	Milk ducts	[177]
LA-PDX2	ILC	PrBC	ER^+^/PR^+^/HER2^−^	Prof. R. Iggo/BCI	Milk ducts	[177]
LA-PDX3	ILC	PrBC	ER^+^/PR^+^/HER2^−^	Prof. R. Iggo/BCI	Milk ducts	[177]
LA-PDX4	ILC	PrBC	ER^+^/PR^+^/HER2^−^	Prof. R. Iggo/BCI	Milk ducts	[177]
BCM-3561	ILC	un	ER^−^/PR^−^/HER2^ENRICHED^	Prof. M.T. Lewis/BCM	Fat pad (mammary)	[178]
BCM-4189	LCIS	MA	ER^−^/PR^−^/HER2^ENRICHED^	Prof. M.T. Lewis/BCM	Fat pad (mammary)	[178]
HCI-005	Mixed NST/ILC	PE	ER^+^/PR^+^/HER2^+^	Prof. A.L. Welm/HCI	Fat pad (mammary)	[179]
HCI-006	Mixed NST/ILC	PE	ER^+^/PR^+^/HER2 ^(n/a)^	Prof. A.L. Welm/HCI	Fat pad (mammary)	[179]
HCI-011	NST	PE	ER^+^/PR^+^/HER2^−^	Prof. A.L. Welm/HCI	Fat pad (mammary)	[179]
HCI-013	ILC	PE	ER^+^/PR^+^/HER2^−^	Prof. A.L. Welm/HCI	Fat pad (mammary)	[179]
HCI-013-EI	ILC	PE	ER^−^/PR^−^/HER2^−^	Prof. A.L. Welm/HCI	Fat pad (mammary)	[168,179]
HCI-014	ILC	PE	ER^−^/PR^−^/HER2^−^	Prof. A.L. Welm/HCI	Fat pad (mammary)	[179]
HCI-018	n/a	BrM	ER^−^/PR^−^/HER2^−^	Prof. A.L. Welm/HCI	Fat pad (mammary)	[179]
HCI-031	ILC/LCIS	PE	ER^−^/PR^−^/HER2^−^	Prof. A.L. Welm/HCI	Fat pad (mammary)	[168]
HCI-031OV	ILC	OvM *	ER^−^/PR^−^/HER2^−^	Prof. A.L. Welm/HCI	Fat pad (mammary)	[168]
WHIM2/5	Mixed NST/ILC	BrM	ER^−^/PR^−^/HER2^−^	Prof. M. Ellis/WUSTL	Fat pad (mammary)	[180]
WHIM9	Mixed NST/ILC	ns	ER^+^/PR^+^/HER2^−^	Prof. M. Ellis/WUSTL	Fat pad (mammary)	[180]
WHIM13	NST (ILC features)	SkR	ER^−^/PR^−^/HER2^−^	Prof. M. Ellis/WUSTL	Fat pad (mammary)	[180]
WHIM20	Mixed NST/ILC	SkC	ER^−^/PR^−^/HER2^+^	Prof. M. Ellis/WUSTL	Fat pad (mammary)	[180]
WHIM23	Mixed NST/ILC	SkC	ER^−^/PR^+^/HER2^−^	Prof. M. Ellis/WUSTL	Fat pad (mammary)	[180]
HBCx-7	ILC	PrBC	ER^−^/PR^−^/HER2^−^	Prof. E. Marangoni, Prof. MF. Poupon/IC	Fat pad (Interscapular)	[172]
HBCx-19	ILC	PrBC	ER^+^/PR^−^/HER2^+^	Prof. E. Marangoni, Prof. MF. Poupon/IC	Fat pad (Interscapular)	[172]
HBCx-36	ILC	PrBC	ER^−^/PR^−^/HER2^+^	Prof. E. Marangoni, Prof. MF. Poupon/IC	Fat pad (Interscapular)	[172]
Met BC 5	ILC	AF	ER^+^/PR^+^/HER2^−^	Prof. R. Clarke/MBRC	Subcutaneous	[174]
Met BC 9	ILC	AF	ER^+^/PR^+^/HER2^−^	Prof. R. Clarke/MBRC	Subcutaneous	[174]
Met BC 11	ILC	AF	ER^+^/PR^+^/HER2^−^	Prof. R. Clarke/MBRC	Subcutaneous	[174]
Met BC 11	ILC	AF	ER^+/^PR^+^/HER2^−^	Prof. R. Clarke/MBRC	Subcutaneous	[174]

The mammary fat pad stroma is a widely used injection site, which is considered a more relevant site for BC engraftment than the skin (Table 6). However, the endogenous mammary epithelium may inhibit the growth of implanted tumor cells [148], and an epithelium-free cleared” fat pad is preferred [181].

Hence, thirty-two stably transplantable xenograft lines have been established by direct injection into the “cleared” mammary gland in the absence of Matrigel, including one LCIS and one ILC [178]. Given the emerging involvement of the HER2 pathway in part of ILCs, global gene expression analyses identified both ILC PDXs as HER2-enriched, yet their corresponding xenografts were HER2^−^ (Table 5, Supplementary File S1). A recent study used 54 BC chunks, including 5 ILCs, to generate PDXs by directly implanting the tumors into the cleared fat pad [163,182]. Interestingly, most ILC PDXs developed in this series harbor *ESR1* alterations commonly implicated as therapy resistance drivers [183]. HCI-018, a brain-derived PDX with a low frequency (<10%) *ESR1*^Y537S^ mutation can only be established in vivo upon E2 supplementation. In HCI-005, E2 induces its growth, and as in the patient, it also metastasizes to the lungs [179]. The HCI-013 was established from a pleural effusion from a 53-year-old woman with metastatic ER^+^/PR^+^/HER2^−^ ILC, stains negative for E-cadherin, cytoplasmic p120 [59], and also depends on exogenous E2 for its in vivo growth. HCI-013EI initially engrafted in ovariectomized mice is an exception as it grew independent of E2. Finally, the TN HCI-031 ILC PDX shows widespread metastasis including uterine horns, stomach, liver, brain, kidney, LN, and spontaneous metastasis to the mouse ovary, a common site of ILC metastases, which generated the secondary HCI-031OV PDX that took nine months to reach a tumor diameter of 2 cm. A series of BC PDXs has been developed by injecting tumor cells into the humanized stroma and endogenous epithelium-free (cleared) mammary fat pads [180,184], including four ILCs. Two of them show high PR levels and carry an activating *ESR1* mutation (WHIM20 *ESR1^Y537S^* and WHIM24 *ESR1^E380Q^*). On the other hand, WHIM9 with a wild-type *ESR1* locus but E2-independent growth and resistance to fulvestrant showed negligible PR levels [180].

A significant challenge in developing ER^+^ PDXs is the substantially low engraftment rate when tumor cells are xenografted subcutaneously or into the mammary fat pad. Notably, very few PDXs are derived from treatment-naïve ER^+^ luminal A ILCs. Direct injection into the murine milk ducts established 21 ER^+^ intraductal PDXs, including 5 ILCs and one mixed subtype. The histological analysis defined four main histopathological patterns: flat, lobular, in situ, and invasive [176]. Markedly, the lobular pattern was characterized by the pagetoid spread of lobular carcinoma in situ (LCIS), associated with intracellular clear mucin-like vacuoles bestowing a signet-ring like appearance of the engrafted tumor cells [176,185]. In another study, four ER^+^ ILC PDXs (Table 5, Supplementary File S1 were generated using the MIND methodology with a 100% engraftment take rate, confirming that ILC tumors engrafted well in the murine milk ducts and gave tumor foci in both secondary and tertiary grafts [177]. Notably, when the same tumor cells were injected in parallel in the milk ducts and subcutaneously into the flanks of NSG mice, the only tumor foci successfully formed was derived from the intraductal ILC models [177]. Therefore, HR^+^ breast tumors, including luminal A, which is the majority of ILCs, can well engraft and propagate in the microenvironment of the mammary milk ducts.

## 7. Other Animals

Sporadic BCs are well studied in dogs and cats, which, like domestic animals, often undergo surgical tumor resections [2,186]. Overall, <10 cases of ILC or LCIS have been described in free-range or domestic animals so far [2]. Over six years, Ressel and colleagues reviewed nearly 4000 canine BCs and identified only three ILC cases [187]. Moreover, three LCIS were identified in macaques in 16 neoplastic mammary gland lesions examined [188]. Recently, immune-deficient zebrafish have been used as a preclinical model to evaluate patient-specific therapy responses [189]. In a recent article, the authors evaluated the engraftment of six PDXs into *prkdc−/−, il2rga−/−* immune-compromised zebrafish, including one therapy-resistant ER^+^/PR^+^/Her2^−^, ILC derived from circulating tumor cells (CTCs) (BRx-07), which harbors activating mutations in *PIK3CA* and *FGFR2* [190,191].

## 8. ILC Challenges

### 8.1. Molecular Mechanisms of ILC Drug Resistance

Endocrine-resistant variants of SUM-44PE and MDA-MB-134-VI have been generated with long-term endocrine treatments in vitro. One of the first in vitro studies generated tamoxifen resistance SUM-44PE cells by increasing concentrations of 4HT [192]. In this model, comparative gene expression analysis with wild-type cells revealed reduced expression of ERα and increased ERRγ (estrogen-related receptor γ) expression, which was, therefore, proposed that may mediate tamoxifen resistance. Moreover, endocrine-resistant variants of MDA-MB-134-VI and SUM-44PE were also generated by long-term maintaining the cells in hormone-deprived conditions. The resulting variants had an increased proliferative response to E2, and in this model, WNT4 expression was driven by activated nuclear factor kappa-B signaling [193]. Additional analysis of the same models subsequently identified activation and induction of several enzymes critical in fatty acid and cholesterol metabolism for energy production. It, therefore, implicated lipid-metabolic processes as drivers of estrogen-independent growth of ILC endocrine-resistant cells [194]. Notably the long-term estrogen-deprived (LTED) ILC cells had higher clonogenic ability compared to their parental cells pointing to the distinct characteristics of resistant cells. In another study, SUM-44PE were cultured without E2 until their growth rate was independent of the exogenous E2 [195]. These models are also characterized by changes in cholesterol biosynthesis enzymes and cholesterol metabolites, suggesting that this pathway could be a therapeutical target in endocrine-resistant ILC [195]. An important ILC resource for studying endocrine resistance has been reported with the discovery of naturally occurring *ESR1*^Y537C^ and *ESR1*^Y537S^ mutations in SUM-44 PE after the in vitro acquisition of resistance to long-term-estrogen deprivation [51]. *ESR1* mutations have been associated with resistance to endocrine therapy, and in this study impacted on *ESR1* binding to the genome and altered the *ESR1* interactome [50,51]. Of note, *ESR1*^Y537S^ was inhibited by fulvestrant but not by 4-OHT, pointing to de novo resistance. Moreover, preclinical in vitro models using the MDA-MB-134-VI cells showed de novo tamoxifen resistance of this particular cell line which overexpress FGFR1 due to high-level amplification of its locus [59]. Clinically, FGFR1 amplifications have been reported as regulators of cell growth and mediators of endocrine therapy resistance [58], and other members of the FGFR family members of tyrosine kinase receptors such as FGFR4 are also overexpressed in ILC resistant cell lines [196].

### 8.2. ILC Tumor Microenvironment

While cell lines, organoids, and animal models provide unique advantages for studying ILC, they are still not ideal for all the basic and preclinical ILC research due to their particular limitations and modeling the tumor microenvironment (TME) presents a unique challenge. For example, 2D and 3D cell line-based models and organoids typically lack the complex ILC tumor–stroma interactions. The ideal ILC model will need to be inclusive of the various cellular components and ECM [197], and in vivo models better mimic this scenario (Table 6). Nevertheless, ILC GEMMs are based on murine TME, and PDXs are developing into immunocompromised hosts (Table 6). To overcome these limitations, we will need, for example, to successfully reconstitute a patient-matched immune system in ILC PDXs.

### 8.3. Modeling ILC Dormancy and Metastasis

As noted earlier, ILC is characterized by distinct clinical features of late recurrence [198]. The comprehensive understanding of the biology of the dormant ILC cells and the molecular mechanisms of metastatic ILC relapse, as described below, is yet largely unclear. Therefore, research on metastatic aspects of lobular carcinomas is of utmost importance because they account for most ILC-related deaths.

In vivo models are of particular interest since they can provide insights into the complex mechanisms of events of cancer progression, including dormancy and metastasis. However, the generation of mouse models that recapitulate the unique features of ILC metastases has been challenging. GEMMs provide a valuable resource for modeling metastasis as they allow both tumor cell-autonomous and stromal influences to be modeled at all stages of the metastatic cascade [199,200]. The early GEMMs of ILC show spread to the gastrointestinal tract and peritoneum in common with what is seen in the human disease and common sites such as lung and bone marrow [25,131]. This is also evident in GEMMs incorporating PI3K pathway mutations [125]. Dormancy has not been explicitly studied using ILC GEMMs as the growth of the primary tumor(s) is typically prohibitive. A significant advance in the field has been the development of orthotopic transplantation of GEMM-derived tumor fragments into syngeneic mice [201], which, when coupled with resection of the primary tumor, provides a much more tractable model for studying dormancy and metastasis and their response to therapy [136,137]. This allows the development of slow-growing metastases without sacrificing the animals due to the rapid growth of the primary tumor. The use of immunocompetent hosts has led to identifying essential roles for the immune system in controlling metastatic outgrowth using the *K14cre;Cdh1^F/F^;Trp53^F/F^* model [138,139]. This approach has been extended to cell lines derived from the KEP model [202,203]. Interestingly, following orthotopic transplantation, the spread was predominantly to the bone, while the KEP cells could also colonize the bone following intracardiac and intratibial transplantation [202].

Metastasis has also been reported in few cases of BC PDX models generated by subcutaneous or fat pad implantation. However, there is a predilection for spread to the lungs and lymph nodes, and typically, they do not fully recapitulate the pattern of metastasis seen in the patient [178,204,205]. In most cases, the metastatic spread is monitored histologically and in a limited number of organs, and more widespread micrometastatic disease may be present. Indeed, in an ILC PDX model (HCI-013) use of Alu-qPCR to detect human sequences suggested widespread dissemination to many different organs, including to the thorax, ovaries, peritoneum, GI tract, and bone, which were not detected histologically or via luciferase imaging (Gomez-Cuadrado, unpublished data). Recently, an ILC PDX model (HCI-031) was established from the pleural fluid of a patient who developed metastases in the fallopian tubes, bones, pleura, liver, and brain. A metastatic variant (HCI-031OV) was then derived from a spontaneous metastasis to the mouse ovary developed from the HCI-031 PDX. This metastatic subline retained the same genomic driver mutations, had similar gene expression profiles as the parental PDX model, and was able to spontaneously metastasize back to the ovary following injection into the cleared fat pad [206].

Typically, xenograft models using human cancer cell lines rarely metastasize in tissues other than the lungs. However, recent studies have shown that intraductal implantation of the MDA-MB-134-VI and SUM-44 PE cell lines leads to their metastatic spread to the leptomeninges, adrenal glands, gastrointestinal tract, ovaries, and the peritoneal cavity—sites of metastasis commonly seen in human disease [160,161,207]. This exciting advance provides a way forward for research into the underlying biology of metastatic disease progression and as a more tractable platform for testing new potential therapies. For example, the use of luciferase imaging of multiple organs demonstrated that lysyl oxidase inhibition reduced the metastatic burden of ILC tumor-bearing animals [160]. These models represent a significant advance in studying the biology of the dormant ILC cells since there is a latency period of up to 5 months before overt metastases are detected. Again, with the caveat that the use of severely immunocompromised mice hampers the study of the immune system’s role in ILC dormancy. Of note, a similar approach was taken with ILC-derived PDX models where spontaneous micro-metastatic spread was found resembling the tumor of origin in various organs, including lungs, bones, brain, and liver following intraductal implantation [31,176].

The study of CTCs and disseminating tumor cells (DTCs) in PDX models provide an additional route to studying metastatic dissemination in a preclinical setting [208]. Of note, ex vivo culture of CTCs from patients with ILC has been reported. When implanted into mice using conventional routes, these CTC-derived xenografts failed to metastasize [191] but grew and metastasized when implanted intraductally [80]. To our knowledge, DTCs studies have not yet been reported for ILC PDX models and, therefore, would be important to model it in future studies.

## 9. Strengths and Limitations of Different ILC Models

Existing ILC models, including cell lines, organoids, and in vivo models, have created unprecedented opportunities for studying the molecular mechanisms of disease progression (Table 6). However, there is concern that only a minority of currently available human BC cell lines used as ILC models are actually of ILC origin. The few ILC models that exist grow very slowly, making them unattractive models. In addition, since they derive from late metastatic disease, they model end-stage ILC having a spectrum of mutations not found in primary ILC (Table 2 and Supplementary File S1). Moreover, ILC lines cannot model the full tumor complexity, and few ILC organoids that exist are not yet comprehensively studied for their endocrine responses (Table 6).

Similarly, while there are adequate in vivo models for other BC subtypes, e.g., TNBC, the field traditionally lacks ER^+^ models, particularly for luminal A subtype that is the majority of ILCs. Direct implantation into the murine milk ducts rather than subcutaneous or fat pad injections recapitulate the complete metastatic capability of ILCs and better reflect the tumor heterogeneity. However, these models lack a functional immune system, limiting their efficacy for testing immunotherapies. Table 6 provides a comprehensive summary of the key advantages and limitations of widely used ILC models presented in this review.

## 10. ILC Initiatives and Repositories of ILC Models

To understand the full potential of ILC models, we should ensure their broad availability and dissemination to the research community. However, the lack of rigorous standards for reporting on PDX models hampered researchers’ finding relevant PDX models and associated data, including ILC PDXs [170]. The European Lobular Breast Cancer Consortium (ELBCC) aims to bridge this gap in translational ILC research and provide an unprecedented clinical impact due to the streamlining of the “from bench-to-bedside” principle to enable uniform diagnosis and tailored treatment for patients with ILC [209]. To this aim LOBSTERPOT, a COST-funded (European Cooperation in Science and Technology) action initiated by the ELBBC, proposes to combine discovery science, translational studies, and clinical implementation to improve the understanding, diagnosis, treatment, and prognosis of women suffering from ILC; https://www.cost.eu/actions/CA19138.

When generating ILC models, emphasis should be placed on quality assurance, careful documentation with associated metadata, and genetic characterization. This documentation will facilitate the data-informed design of the appropriate ILC model to examine preclinical drug responses of primary tumors and metastases. Although several repositories have been developed recently (Appendix A), a centralized specific ILC public model repository is required for the reporting, quality control, and standardized use of cell lines, organoids, and ILC animal models [170]. To this end, it will be crucial to share knowledge on the appropriate use of ILC models for immune assessments, dormancy, or treatment of established tumors and metastases. Finally, limitations to each model (e.g., limited metastatic potential, the time needed from initial engraftment to micro/macro metastasis) should also be thoroughly addressed.

## 11. Discussion

ILC models have proved useful for preclinical studies and have provided findings that can positively affect clinical management. However, as noted earlier in this review, although valuable, the few available ILC models all have limitations. For example, cell lines remain a powerful tool for BC research however, there is an increased risk of in vitro genomic evolution [30]. The number of available ILC models remains limited, and the widely used models do not represent primary nonmetastatic ILCs with genetic repertoire such as wild-type p53, activating *PIK3CA/HER2* mutation, and low-grade treatment-naïve ER^+^ luminal A ILCs. Given the challenges in developing new ILC cell lines, we characterize current models in the ILC Cell Line Encyclopedia (ICLE). The ongoing ICLE project is mirrored on the successful Cancer Cell Line Encyclopedia (CCLE) [now named DepMap—https://depmap.org/portal/], which unfortunately included only two ILC cell line models. Using similar approaches, we plan to integrate the ICLE cell line results with CCLE. We are performing comprehensively molecular profiling of a total of 17 ILC and ILC-like cell lines, yet preliminary data show that they have genetic features of ILC such as mutation of *CDH1*. The final product will be a publicly available website with comprehensive information about DNA, RNA, and protein expression in a large panel of BC cell lines. We will also combine this with limited phenotypic data to ensure that researchers can readily find the most appropriate cell line model for their research.

Significant advances have been made in the ex vivo BC modeling; however, due to the loss of E-cadherin expression, ILC has defective adherens junctions, which impedes their proper 3D formation, development, and expansion of non-coherent ILC organoids, and only a few models have been successfully propagated. We expect an increased number of well-characterized ILC PDOs in the near future.

Modelling ILC with GEMMs, where the mouse germline can be readily manipulated to induce overexpression or knockout of target genes, is an essential tool for understanding ILC biology [210]. However, tumors typically harbor numerous driver lesions, affecting their sensitivity to treatments [211]. As such, it remains crucial to establish complex mouse models incorporating multiple driver genes that are frequently encountered together in ILCs. Moreover, current ILC-specific trials (NCT03620643 and NCT04551495) are conceptually based on functional findings from the conditional ILC mouse models.

PDXs largely recapitulate the polygenic architecture of human tumors and closely resemble patient tumor molecular and histological features [30,168]. The realization that cell-cell and cell-matrix interactions within the complex tumor environment regulate primary tumor growth, metastatic spread, and response to therapy provides potential new ILC therapeutic opportunities [160,212,213,214]. Similarly, as we now understand more about the unique ILC tumor microenvironment, it will be necessary to model the different immune cell populations, cancer-associated fibroblasts, and matrix components [29,160,213,215]. Moreover, the establishment of PDX models from unique ILC metastatic sites such as leptomeninges and ovaries would provide additional information on how the tumor-related metastatic niche controls the outgrowth of disseminated ILC cells. ILC post-mortem tissue donation programs such as the recent interventional (Clinical Trial) named UPTIDER (https://clinicaltrials.gov/ct2/show/NCT04531696) will further support basic and translational research to model ILC dormancy and metastasis, to understand drug resistance, and develop new effective therapies [216]. This is particularly important in ILC progression often characterized by an increased risk of late recurrence, attributed to the reawakening of dormant disseminated tumor cells after prolonged periods. We anticipate that PDX models will help us better understand the mechanism of anti-endocrine therapies studies and the exquisite ILC sensitivity to estrogens plus progesterone treatments [217]. In that direction, we envisage that the intraductal model will help us study early in situ lesions that have a repertoire of somatic genetic alterations similar to that of ILCs [218]. Moreover, rare ILC cases should now be studied, including male ILC, which needs further investigation [219,220,221]. To our knowledge, there is only one unpublished male PDO (Table 3); yet, it has not been used to develop a PDX and needs further characterization.

Together, the potential use of humanized ILC mouse models may improve the prediction of the therapeutic efficacy of novel agents. The further development and refinement of the complex ILC animal models will provide understanding and identification of metastatic pathways, new therapeutic targets, and conceivably personalized cancer therapy.

## 12. Conclusions

We lack preclinical ILC models to develop new effective treatments. We envisage that lobular research initiatives, such as LOBSTERPOT-CA19138 action, will play an essential role in understanding the existing in vitro and in vivo ILC models and developing new models. Moreover, the growing knowledge about the challenges and the opportunities of ILC models that we describe in this review, together with the development of early- and metastatic-stage clinical trials specifically for patients with E-cadherin-deficient tumors, presents an exciting opportunity for further improving and expanding ILC models.

## Figures and Tables

**Table 1:** Milestones timeline of key lobular models development and their applications. Chronological list of the key ILC models leading to existing lobular models and their established and future applications. Abbreviations: P, proliferation; TN, triple negative; GEM model, genetically engineered mouse model; LCIS, lobular carcinoma in situ; I, invasion; TP, tumor progression; EnR, endocrine response, and resistance; HoR, hormone responsiveness; C, chemoresistance; D, dormancy; M, metastasis; MM134, MDA-MB-134-VI; SUM44, SUM-44PE; PMID, PubMed identifier; PILC, pleomorphic ILC; SR, signet ring morphology; T, targetoid; R, round; SF, single-cell-file pattern. Color code: green, cell lines in vitro; grey, GEMMs; orange, organoids; purple, xenografts, and PDXs.

Year	Model	Significance	Morphological Characteristics	Applications	PMID
1974	MDA-MB-134-VI	First ILC cell line - expansion/characterization (in vitro)	R, SF	P, EnR, HoR, DrR	4412247
1978	SUM-44PE	ILC cell line - expansion/characterization (in vitro)	R, SF	P, EnR, HoR, DrR	8425198
2006	*Cdh1*; *Trp53*	First GEM ILC model Rational: *CDH1* is an ILC hallmark	PILC	LCIS, P, TP, I, DrR, D, M	17097565
2008	SUM-44PE variants	Studies on endocrine resistance (in vitro)	R, SF	P, EnR	18974135
2009 2012	IPH-926	First well characterized TN ILC cell line	R, SF	P, DrR	19191266 22945757
2011	ILC PDXs (cleared mammary fat pad)	First ILC PDXs	LCIS, PILC, SR, SF	P, TP, EnR, HoR, DrR, D, M	22019887
2016	*Cdh1*; *Pten*	GEM ILC model. Rational: PTEN loss found in ~10% of ILCs	cILC	LCIS, P, TP, I, EnR, HoR, DrR, D, M	27524621
2016 2018	ILC PDXs (murine milk ducts)	First intraductal ILC PDXs	LCIS, PILC, SR, SF	LCIS, P, TP, I, EnR, HoR, DrR, D, M	30430577 19191266
2018	Breast Cancer Organoids	First ILC organoids	SR	P, EnR, HoR, DrR	29224780
2018	*Cdh1*; *Pik3ca*	GEM ILC model. Rational: PIK3CA mutations found in ~40% of ILCs	cILC, SF, T	LCIS, P, TP, I, EnR, HoR, DrR, D, M	30332649
2021	ILC xenografts (murine milk ducts)	First intraductal SUM-44PE and MDA-MB-134-VI xenografts	LCIS, PILC, SR, SF	LCIS, P, TP, I, EnR, HoR, DrR, D, M	33616307

**Table 2:** ILC and ILC-like cell lines. Information was collected from published literature. Widely used ILC and ILC-like breast cancer cell lines. Abbreviations: ΜA, malignant ascites; PF, pericardial fluid; BM, bone metastasis; OvM, ovarian metastases; ER, estrogen receptor; NST, non-special type; PE, pleural effusion; BmM, bone marrow metastases; n/a, not available; Ref, reference(s); *, premature termination (stop) codon; npy, not published yet. See also expanded table at Supplemental File S1.

Name	Tissue	Tumor	Biomarker	E-Cadherin/*CDH1*	Morphology	Ref.
**ILC Cell Lines**						
SUM-44 PE	PE	ILC	ER^+^, PR^low^, HER2^−^	p.F423LfsX8	Rounded	[33]
IPH-926	MA	ILC	ER^−^, PR^−^, HER2^−^	p.V82fsX93	Rounded	[34]
MDA-MB-134-VI	PE	NST	ER^+^, PR^−^, HER2^−^	p.L230EfsX4	Rounded	[33]
MDA-MB-330	PE	ILC	ER^+^/^−^, PR^−^, HER2^+^	wt	Rounded	[35]
UACC-3133	PE	ILC	ER^low^, PR^−^, HER2^+^	n/a	n/a	[36]
MA-11	BmM	ILC	ER^−^, PR^−^, HER2^−^	n/a	Rounded	[37]
WCRC-25	PE	ILC	ER^−^, PR^−^, HER2^−^	p.Q706 *	Rounded	npy
**ILC-Like Cell Lines**						
BCK4	PE	ILC (mucinous)	ER^+^, PR^+^, HER2^−^	n/a	Rounded	[38]
MDA-MB-453	PF	n/a	ER^−^, PR^−^, HER2^+^	p.W638X	Rounded	[39]
MDA-MB-468	PE	n/a	ER^−^, PR^−^, HER2^−^	wt	Rounded	[39]
CAMA-1	PE	Solid	ER^+^, PR^+^, HER2^−^	p.Y523_G571del	Rounded	[39]
SK-BR-3	PE	n/a	ER^−^, PR^−^, HER2^+^	c.1_1936del1936	Rounded	[35]
SK-BR-5	n/a	n/a	ER^−^, PR^−^, HER2^+^	p.I178TfsX32	n/a	[40]
EVSA-T	AF	n/a	ER^−^, PR^−^	p.V216_T229del	Rounded	[41]
CAL-148	PE	n/a	ER^−^, PR^−^, HER2^−^	D402N, deep deletion	n/a	[42]
ZR-75-30	AF	NST	ER^+^, PR^−^, HER2^+^	p.Glu243Ter-p.E243X	Rounded	[43]
HCC2218	PBC	NST	ER^−^, PR^−^, HER2^+^	c.1-832del	Rounded	[44]
600MPE	PE	NST	n/a	p.Y380_K440del	Rounded	[45]
BT549	n/a	Papillary	ER^−^, PR^−^, HER2^−^	n/a	Rounded	[46]
MA-11	n/a	ILC and tubular	ER^−^, PR^−^, HER2^−^	wt	Rounded	[37]
OCUB-1F	PE	n/a	ER^−^, PR^−^	p.Val17fs*1	Rounded	[47]

**Table 3:** ILC Patient-Derived Organoids. Expanded table at Supplemental File S1. Abbreviations: HI, Hubrecht Institute; ICR, The Institute of Cancer Research; UMC, University Medical Center Utrecht; UDL = UMCU Derksen Lab; PILC, pleomorphic ILC; Ref, reference(s); n/a, not available; npy, not published yet.

Name	Type	Clinical (Biomarker)	Organoids (Biomarkers)	E-Cadherin/*CDH1*	Laboratory/Institute	Ref.
**Human ILC Organoids (female)**						
T35	ILC	ER^+^, PR^+^, HER2^−^	ER^+^, PR^−^, HER2^+^	n/a	Prof. Hans Clevers/HI	[117]
T66	ILC	ER^+^, PR^+^, HER2^+^	ER^+^, PR^+^, HER2^+^	n/a	Prof. Hans Clevers/HI	[117]
T74	ILC (apocrine?)	ER^+^, PR^+^, HER2^−^	ER^+^, PR^+^, HER2^−^	n/a	Prof. Hans Clevers/HI	[117]
T105	ILC	ER^+^, PR^+^, HER2^−^	ER^+^, PR^+^, HER2^−^	n/a	Prof. Hans Clevers/HI	[117]
P008	ILC	ER^+^, PR^−^, HER2^−^	ER^−^, PR^−^, HER2^−^	p.(Ser180Tyr)	Prof. Clare Isacke/ICR	npy
KCL320	ILC	ER^+^, PR^+^, HER2^−^	ER^−^, PR^−^, HER2^−^	splice variant g.68823627G>A	Prof. Clare Isacke/ICR	npy
**Human ILC Organoids (male)**						
UDL-MBC6	ILC	n/a	n/a	c.85del p.(His29fs)	Prof. Patrick WB Derksen/UMC Prof. Van Diest/UMC	npy
**Mouse primary ILC**						
UDL-WEP9	PILC	ER^−^, PR^−^, Her2^−^	ER^−^, PR^−^, Her2^−^	null	Prof. Patrick WB Derksen/UMC	npy
UDL-WEP10	PILC	ER^−^, PR^−^, Her2^−^	ER^−^, PR^−^, Her2^−^	null	Prof. Patrick WB Derksen/ UMC	npy

**Table 4:** ILC GEMMs. Information was collected from published literature. Abbreviations: Tg, transgenic; CRISPR, Clustered Regularly Interspaced Short Palindromic Repeats; Cas9, CRISPR-associated protein 9; GEMM-ESC, genetically engineered mouse model-embryonic stem cell; ILC, invasive lobular carcinoma; ER, estrogen receptor; U of T, University of Toronto; NKI, Netherlands Cancer Institute; n/t, not tested; n/s, not specified; Ref, reference(s). See also expanded Supplemental File S1.

Deletion/Activation	System	Primary Tumor	ER	Tumor Onset (Weeks)	Laboratory/Institute	Ref.
*Cdh1; Tp53*	Tg	Pleomorphic ILC	Neg.	20–32	Prof. Jos Jonkers/NKI	[25]
*Cdh1; Pten*	Tg	Classical ILC-like features	Pos.	8–16	Prof. Jos Jonkers/NKI	[125]
*Cdh1 and Pik3ca*	Tg	Immune-related ILC-like	Pos.	5–12	Prof. Sean E. Egan/U of T	[126]
*Cdh1 and Pten*	CRISPR/Cas9	Unknown-ILC histology	n/t	28	Prof. Jos Jonkers/NKI	[127]
*Cdh1 and AKT^E17K^*	GEMM-ESC	Typical ILC histology	n/t	n/s	Prof. Jos Jonkers/NKI	[127]
*Cdh1 and Myh9*	CRISPR/Cas9	Classical ILC-like features	n/t	n/s	Prof. Jos Jonkers/NKI	[128]
*Cdh1; t-ASPP2*	GEMM-ESC	Classical ILC-like features	n/t	9–15	Prof. Jos Jonkers/NKI	[128,129]
*Cdh1; Pten; t-ASPP2*	GEMM-ESC/Tg	Classical ILC-like features	n/t	5–9	Prof. Jos Jonkers/NKI	[129]
*Cdh1; t-MYPT1*	GEMM-ESC	Classical ILC-like features	n/t	10–16	Prof. Jos Jonkers/NKI	[128,129]
*Cdh1; Pten; t-MYPT1*	GEMM-ESC/Tg	Classical ILC-like features	n/t	5–8	Prof. Jos Jonkers/NKI	[129]
*Cdh1; Trps1*	GEMM-ESC	Classical ILC-like features	n/t	76	Prof. Jos Jonkers/NKI	[130]

**Table 6:** Experimental ILC models. Abbreviations: SM, spontaneous metastasis; EM, experimental metastasis; GEMMs, genetically engineered mouse models; PDXs, Patient-derived xenografts; E2, estradiol. Color code: green, cell lines; orange, organoids; grey, GEMMs; purple, xenografts, and PDXs; white, implantation sites.

ILC Models	Major Experimental Pros	Major Experimental Cons	Ref.
**Cell lines (in vitro)**	Easy to work with Well characterized High-throughput drug screens	A high degree of variation across cell line strains Lack of tumor heterogeneity Lack of the complex ILC–stroma interactions Not fully represent human cancers complexity	[33,34,36,37,39,63]
**Organoids**	Captures complex 3D cellular interactions	Lack of the complex ILC–stroma interactions Variations in culture conditions Overgrowth by healthy epithelial organoids Survival for a relatively short period compared to in vivo models	[117]
**GEMMs**	Captures complex ILC tumor–stroma interactions Easily manipulated (mouse germline) to induce overexpression or knockout of target genes and study them in the context of an intact mammalian organism Spontaneous metastasis in clinical relevant sites/organs Murine immune-proficient	Time-consuming Fast growth of the tumor cells at the primary site Development of mammary tumors in multiple glands Limited metastatic capacity (e.g., lack of leptomeninx and ovarian metastases)	[25,125,126]
**Cell lines (in vivo–xenografts)**	Easy to work with Well-characterized cell lines Studies on metastasis Studies on endocrine resistance in vivo	Time-consuming Lack of tumor heterogeneity Immune deficient	[38,53,154,160,193]
**PDXs**	Maintain clonal diversity of the original tumors Serial transplantation Preclinical testing of ILC therapies Studies on metastasis Studies on endocrine resistance in vivo	Large amount of resources Time-consuming Immune deficient Limited ILC tissue quantity	[34,153,154,160,163,172,174,175,176,178,179]
**Implantation Sites**			
Subcutaneous (SM)	Easy to perform	Low take rate Ectopic implantation Circumvent the early steps of the metastatic cascade No recognizable architectural ILC features Need for exogenous E2 supplementation (pre-menopause levels) Rarely metastatic Immune deficient	[172,174,175]
Mammary fat pad (SM)	Orthotopic The anatomical site reflects human breast	Low take rate Circumvent early steps of tumor progression (e.g., LCIS) Immune deficient Low take rate Need for exogenous E2 supplementation (pre-menopause levels)	[178,179]
Injection into the milk ducts (SM)	Transplantation in the proper anatomical context where tumor arises High take rate Exogenous E2 supplementation is not a prerequisite for ILC in vivo establishment ILC histologies Recapitulates the complete tumor progression and metastatic cascade Spontaneous metastasis in clinically relevant sites/organs	Intraductal injection skills Immune deficient	[154,160,176]
Tail vein (EM)	Easy to perform Fast systemic distribution of cells to various organs Absence of primary tumor	Circumvent early steps of the metastatic cascade A high number of cells injected directly into the circulation and rapidly colonize an organ/tissue Mainly lung metastasis	[207]
Intracardiac (EM)	Fast systemic distribution of cells to various organs Absence of primary tumor	Circumvent early steps of the metastatic cascade A high number of cells injected directly into the circulation and rapidly colonize an organ/tissue Mainly brain and bone metastasis Invasive technique	[207]

## Supplementary Materials

The following are available online at https://www.mdpi.com/article/10.3390/cancers13215396/s1, Supplementary File S1: Comprehensive description of ILC models.

## Abbreviations

ER	Estrogen Receptor
PR	Progesterone Receptor
HER2	Human Epidermal Growth Factor Receptor 2
PDX	Patient-Derived Xenograft
PDO	Patient-Derived Organoid
GEMMs	Genetically Engineered Mouse Models
Xenograft	Human-derived cells and tissues transplanted into a recipient of another species (mice, rats, zebrafish, etc.)
CaMa	Cancer Mammary
NSG	NOD.Cg-*Prkdc^scid^ Il2rg^tm1Wjl^*/SzJ
ELBCC	The European Lobular Breast Cancer Consortium
COSMIC	Catalogue Omf Somatic Mutations In Cancer
ECM	Extracellular matrix
CTC	Circulating Tumor Cell
DTC	Disseminating Tumor Cell
HCMI	Human Cancer Models Initiative
KCLB	Korean Cell Line Bank
CCLE	Cancer Cell Line Encyclopedia
RIKEN	Rikagaku Kenkyūjo Cell Bank
ATCC	American Type Culture Collection

## Appendix A

### Appendix A.1. Initiatives and Models Repositories

1. Platform to bring together discovery scientists, translational researchers, and clinical experts, with the ultimate goal to improve the understanding, diagnosis, treatment, and prognosis of women who have Invasive Lobular Breast Cancer—http://www.elbcc.org/
2. Lobular Breast Cancer: Discovery Science, Translational Goals, Clinical Impact—https://www.cost.eu/actions/CA19138/#tabs|Name:overview
3. The Lobular Breast Cancer Alliance (LBCA) has compiled a reference list of research publications related to lobular breast cancer—https://lobularbreastcancer.org/
4. Patient-Derived Xenograft and Advanced In Vivo Models (PDX-AIM) Core of Baylor College of Medicine. PDX Portal is designed to allow investigators to browse the various organ site-specific PDX collections—https://pdxportal.research.bcm.edu/pdxportal/?dswid=8822
5. PDX Finder—a comprehensive open global catalogue of PDX models and their associated data across resources—https://www.pdxfinder.org/
6. The PDXNet comprises six PDX Development and Trial Centers (PDTCs) and the PDX Data Commons and Coordinating Center (PDCCC), and is working with the National Cancer Institute to translate PDX studies to clinical trials.
7. EurOPDX Consortium is an initiative of translational and clinical researchers counting 18 not-for-profit cancer centers and universities as members across Europe and the US, https://www.europdx.eu/
8. The Human and Mouse Linked Evaluation of Tumors (HAMLET) Core developed by Prof. Matthew Ellis, M.D., Ph.D., and Shunqiang Li (Director: Shunqiang Li, Ph.D.—https://digitalcommons.wustl.edu/hamlet/
9. Public Repository of Xenografts (ProXe) is an open-source website designed to disseminate information relevant to patient-derived xenografts (PDXs) and PDXs from patients with leukemia or lymphoma by the Weinstock Laboratory. Many models are being licensed to the Jackson Laboratories for industry-scale purposes, including distribution on a fee-for-service basis.
10. Cell Model Passports—a hub for clinical, genetic, and functional datasets of preclinical cancer models, https://cellmodelpassports.sanger.ac.uk/ [222].
11. NCI Patient-Derived Models Repository (PDMR)—https://pdmr.cancer.gov/

### Appendix A.2. Companies

1. Novartis Institutes for Biomedical Research PDX Encyclopedia (NIBR PDXE), an industry-led initiative that includes approximately 1000 models
2. Horizon (The WHIM, Washington University Human-in-Mouse, collection developed by Shunqiang Li and Matthew Ellis at Washington University and published in Cell Reports). ILC models are reported in Table 5 and Supplementary File S1.4.
3. XenOPAT (a spin-off of the Institute of Biomedical Research (IDIBELL), the Catalan Institute of Oncology (ICO) and then University Hospital of Bellvitge).
4. Crown Bioscience Inc., http://hubase.crownbio.com/
5. Champions TumorGraft^®^ Database—https://database.championsoncology.com/models/filter. *Run PDX studies (in-house) to test experimental oncology compounds.
6. Charles River has more than 400 fully characterized proprietary patient-derived xenografts (PDXs), representing major histotypes and tumors and providing extensive background and characterization. No ILC model was found in the database.
