# Supplementary material for: Atlas of Lobular Breast Cancer Models: Challenges and Strategic Directions
Source: Cancers (Basel). 2021 Oct 27;13(21):5396. doi: 10.3390/cancers13215396 (PMC8582475; doi:10.3390/cancers13215396)
Supplement: Supplementary file 1 [file cancers-13-05396-s001.zip › 20210924_Appendix B.pdf]

Appendix B

Brightfield Microscopy Image Gallery of ILC Cell line Models described in Table 2 and Suppl. S1.1

SUM-44 PE

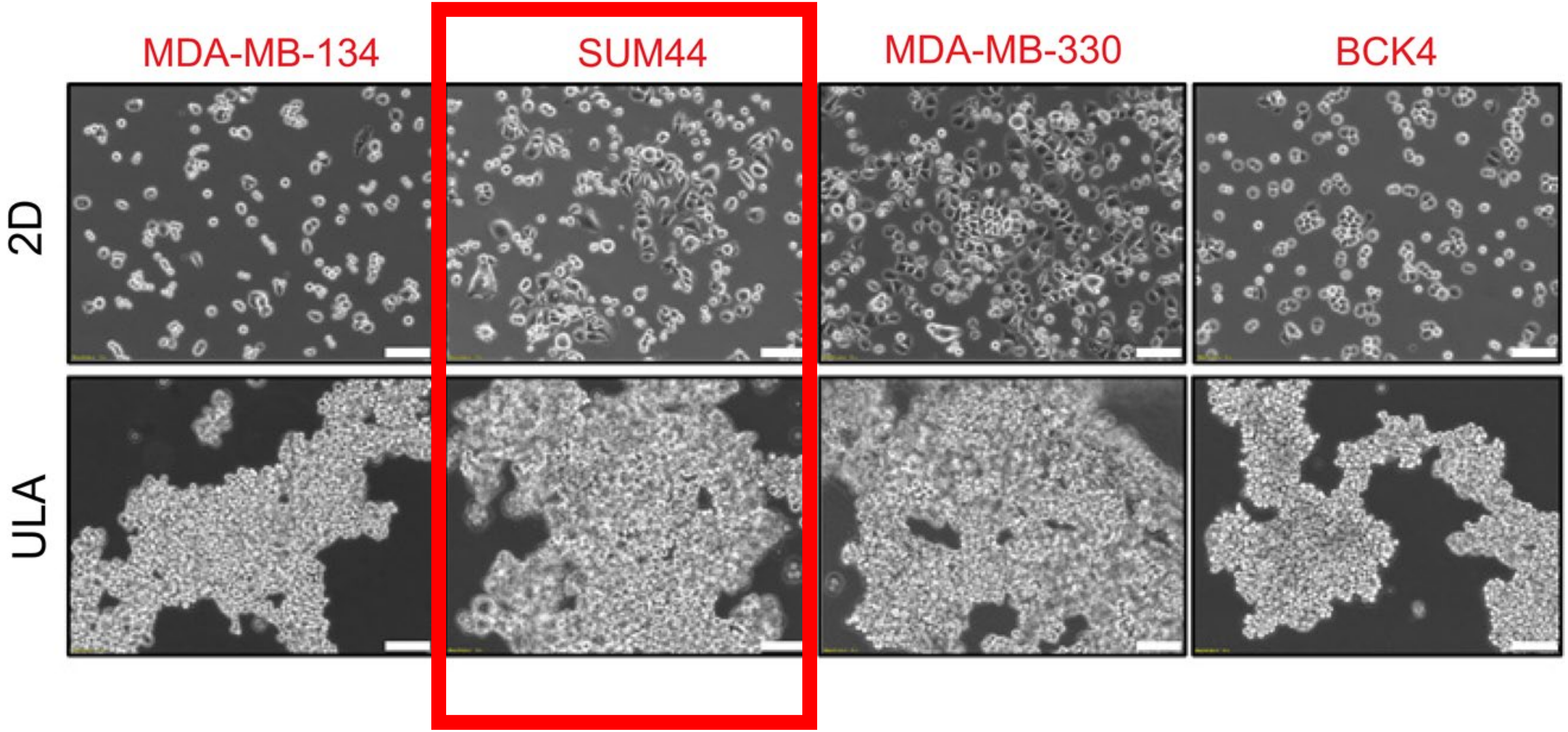

Supplementary Figure 1. Source: https://pubmed.ncbi.nlm.nih.gov/30228172/

IPH-926

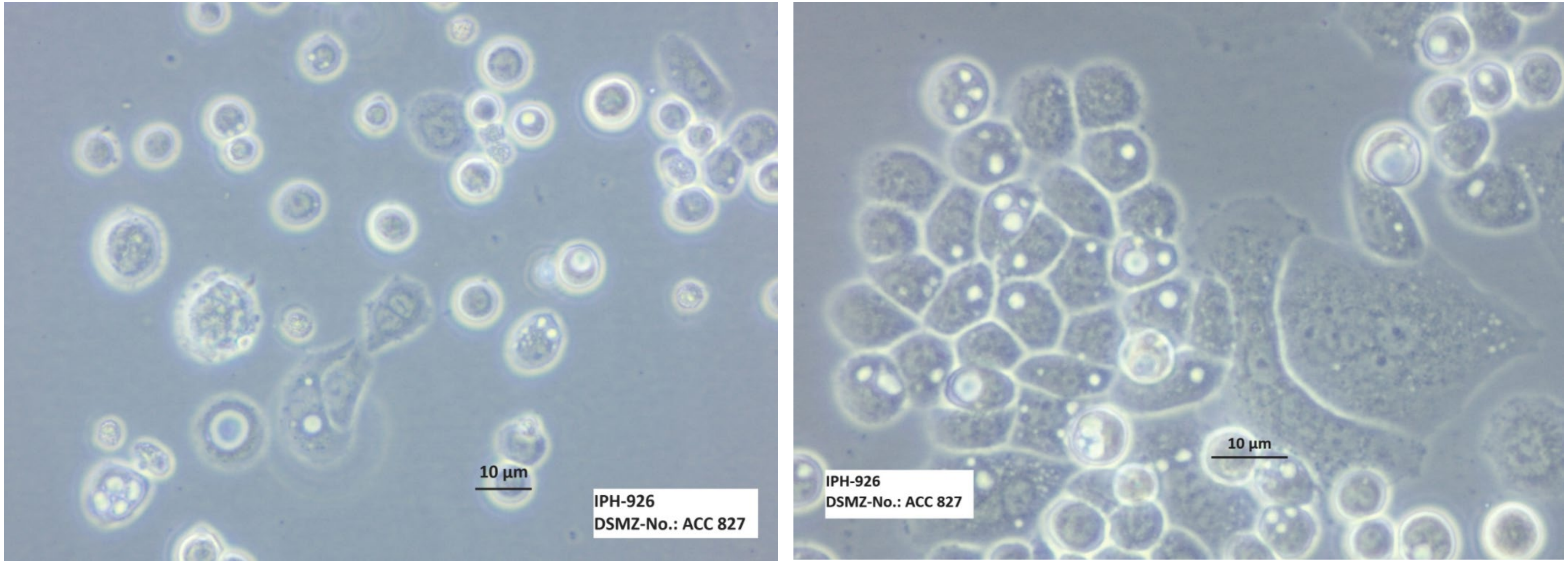

Supplementary Figure 2. Source: DSMZ

MDA-MB-134 PE

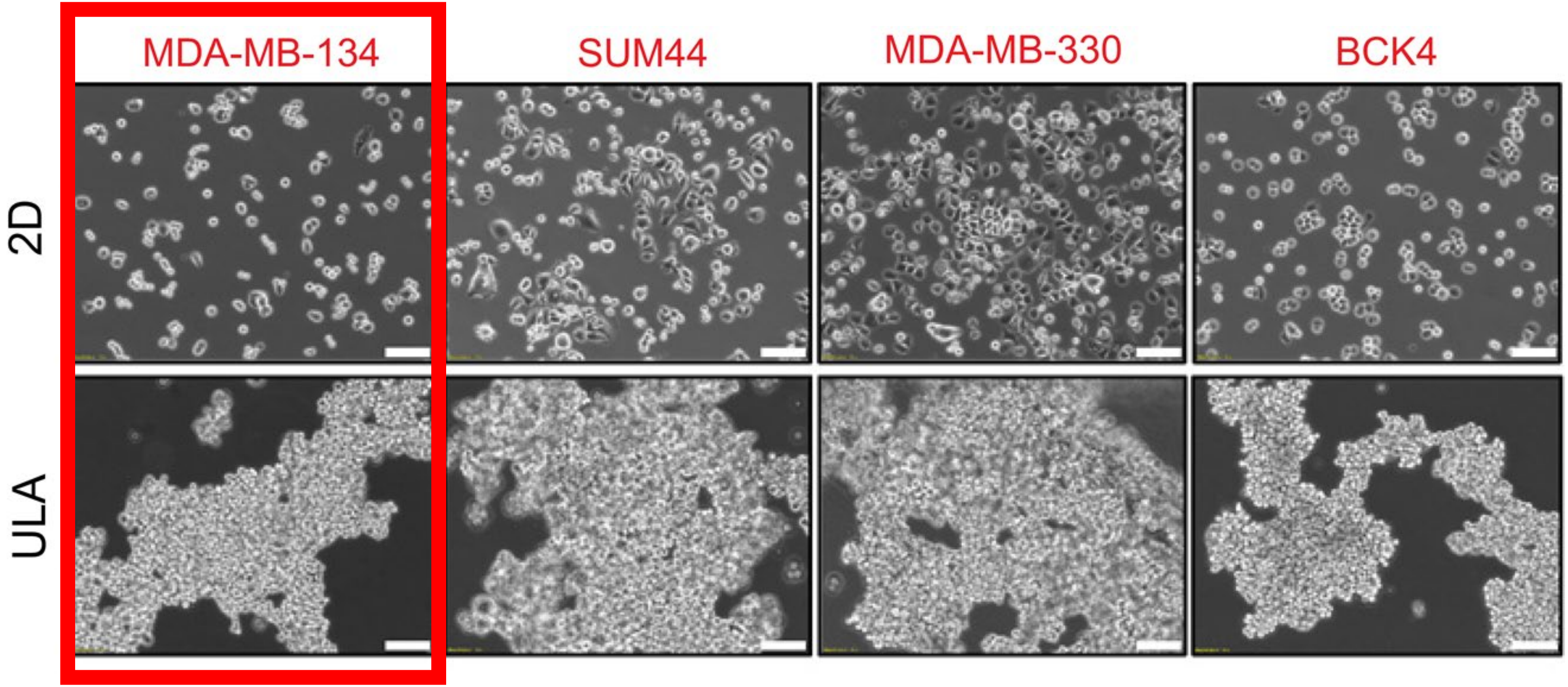

Supplementary Figure 3. Source: https://pubmed.ncbi.nlm.nih.gov/30228172/

MDA-MB-134 PE

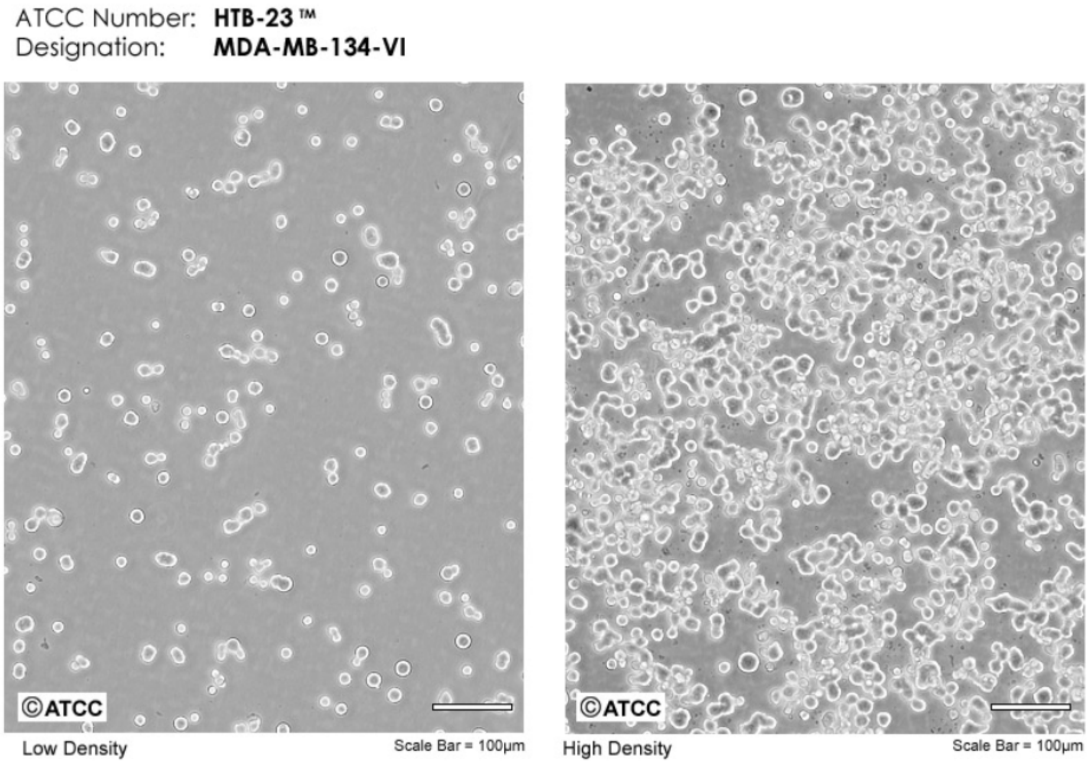

Supplementary Figure 4. Source: ATCC

MDA-MB-330

ATCC Number: **HTB-127**
Designation: **D341 Med**

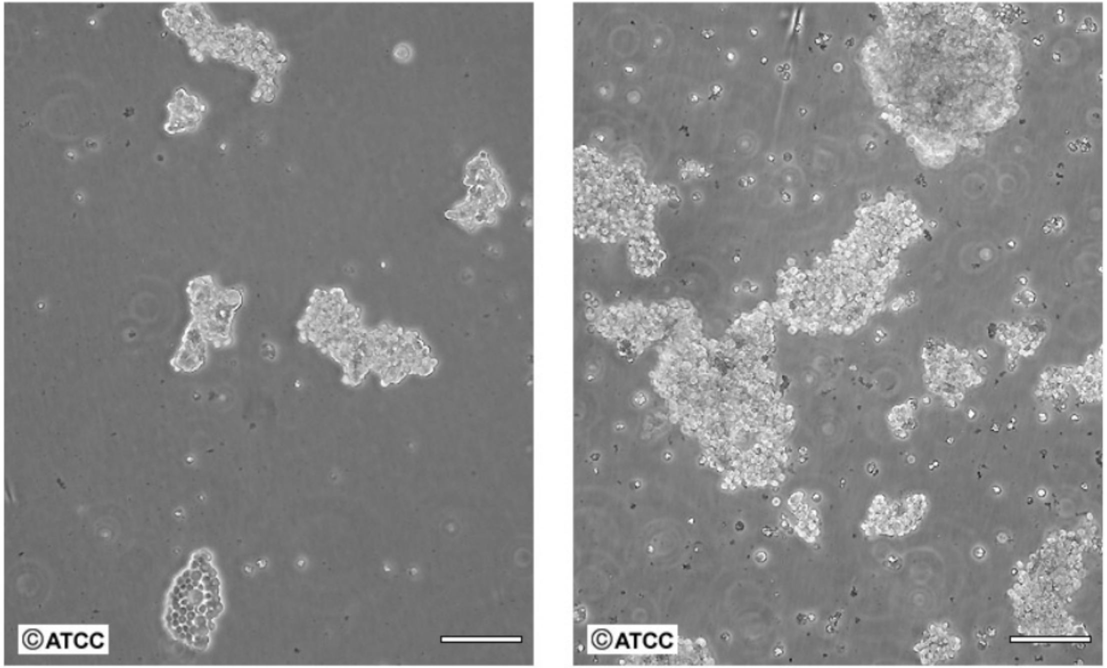

Low Density Scale Bar = 100µm
High Density Scale Bar = 100µm

Supplementary Figure 5. Source: ATCC

UACC-3133

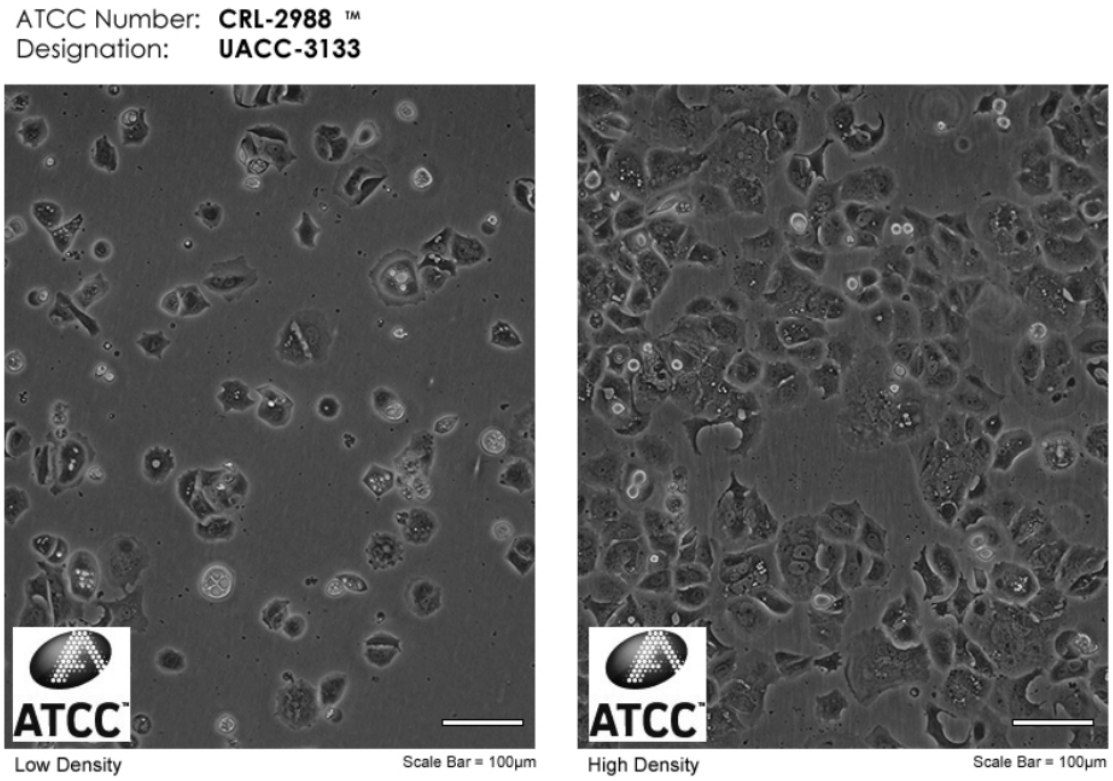

Supplementary Figure 6. Source: ATCC

HCC-2185*

*Discontinued in ATCC

Supplementary Figure 7. Source: ATCC

BCK-4

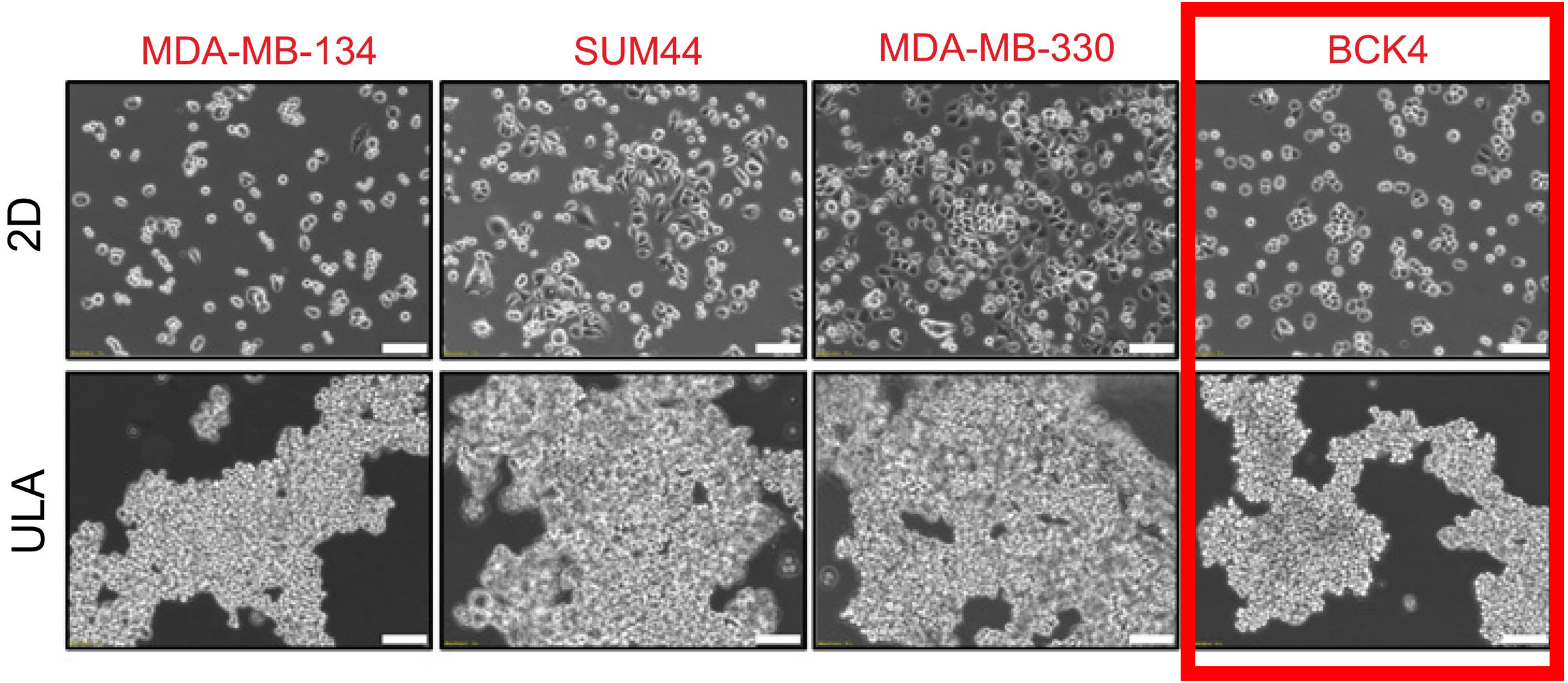

Supplementary Figure 8. Source: https://pubmed.ncbi.nlm.nih.gov/30228172/

MDA-MB-453

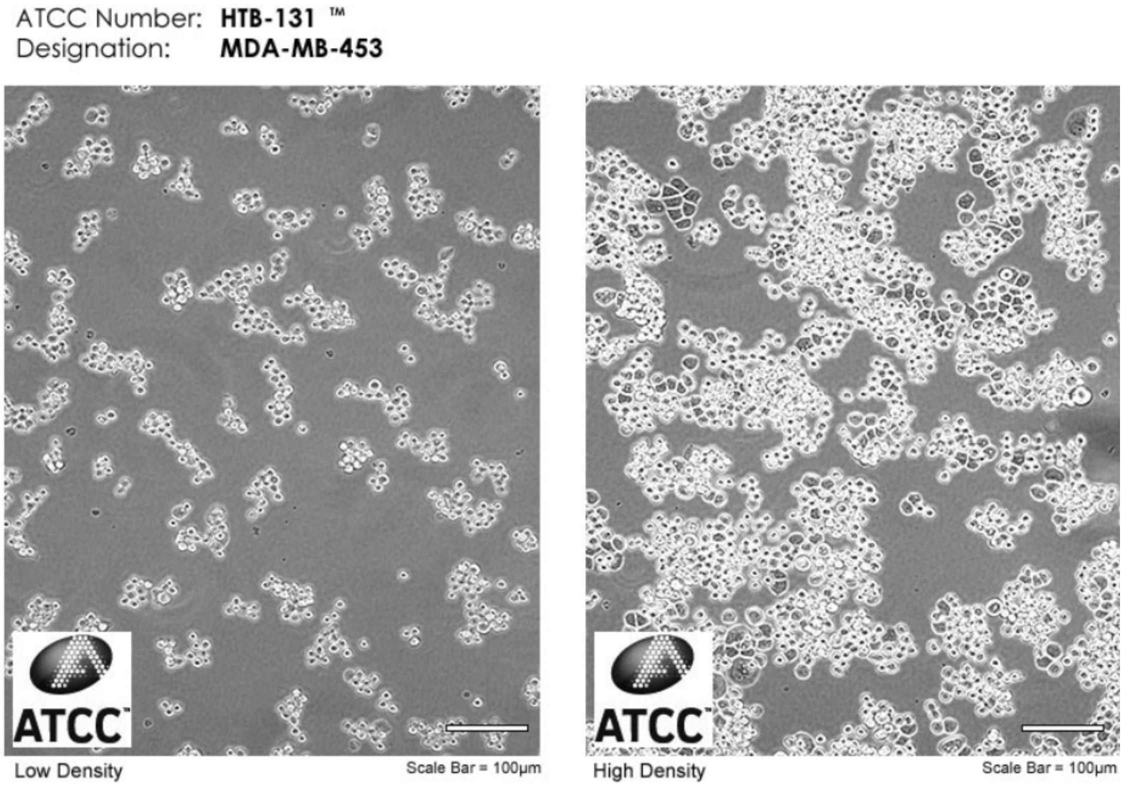

Supplementary Figure 9. Source: ATCC

EVSA-T

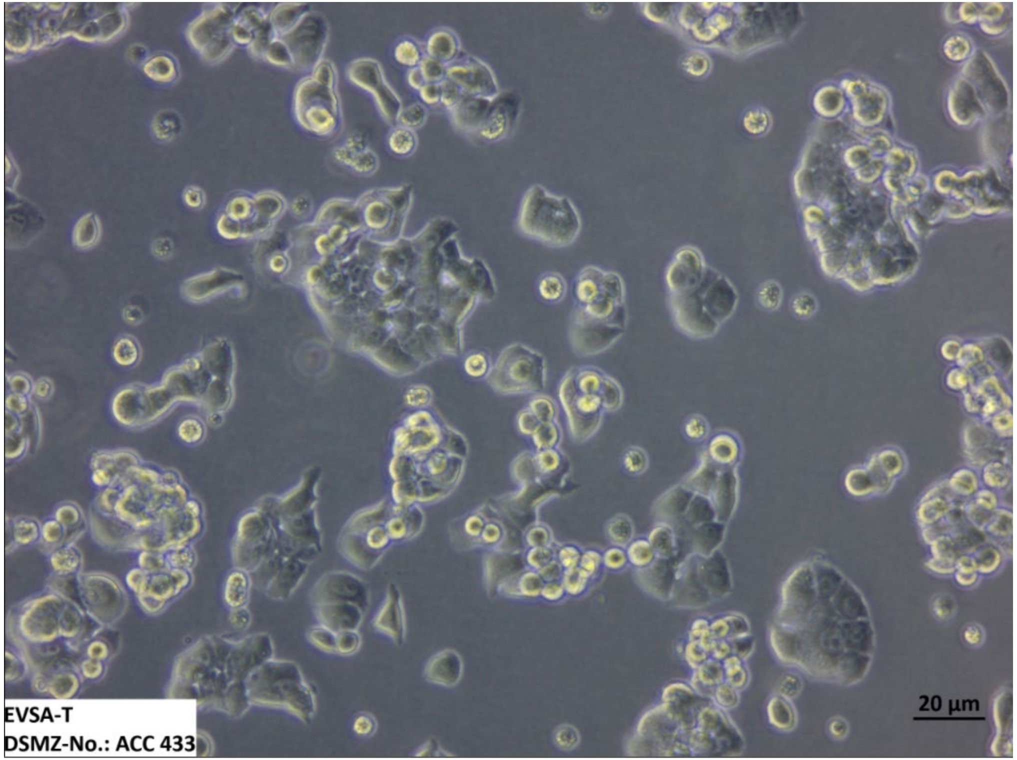

Supplementary Figure 10. Source: ATCC

ZR-75-30

ATCC Number: **CRL-1504**
Designation: **ZR-75-30**

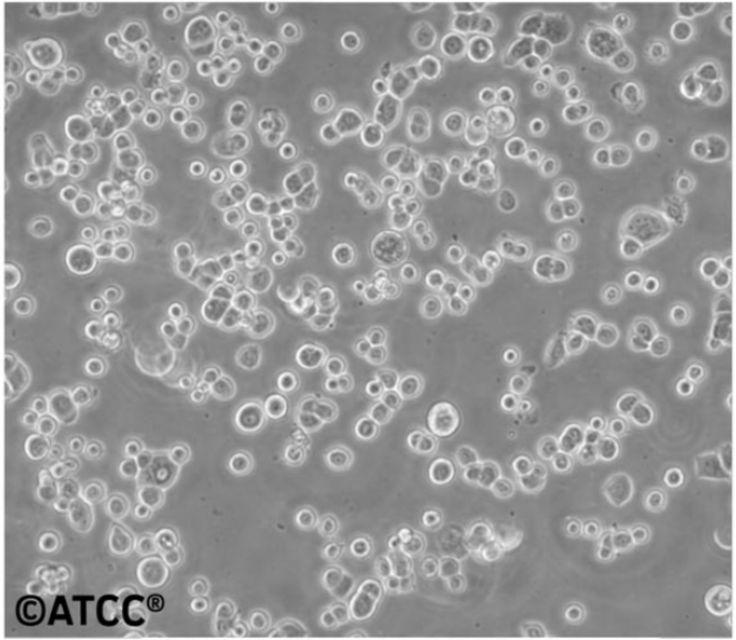

Low Density

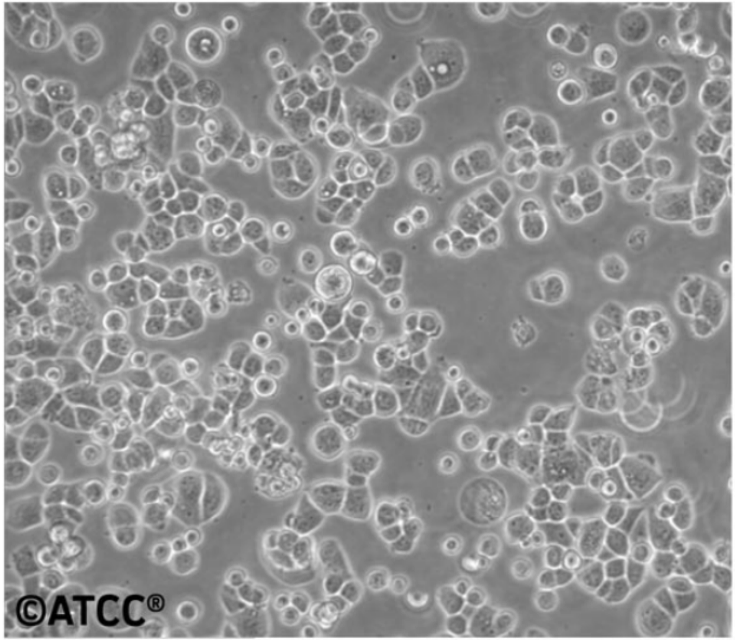

High Density

Supplementary Figure 11. Source: ATCC

CAMA-1

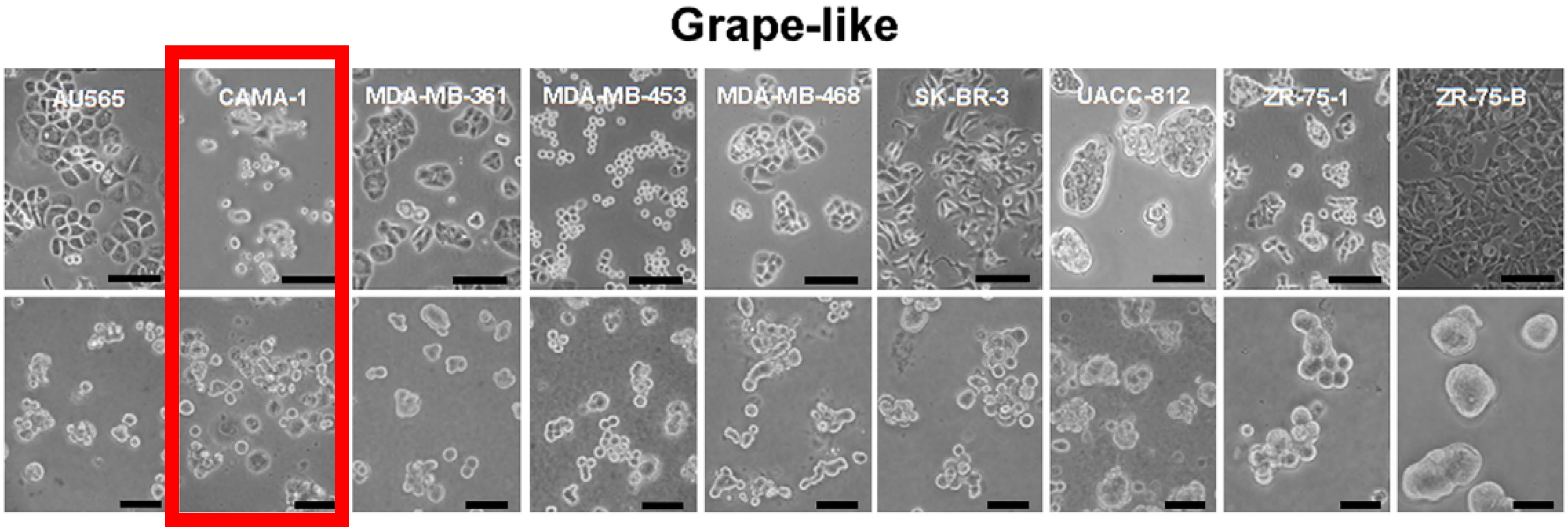

Supplementary Figure 12. Source: https://doi.org/10.1016/j.molonc.2007.02.004

HCC2218

ATCC Number: **CRL-2343**
Designation: **HCC2218**

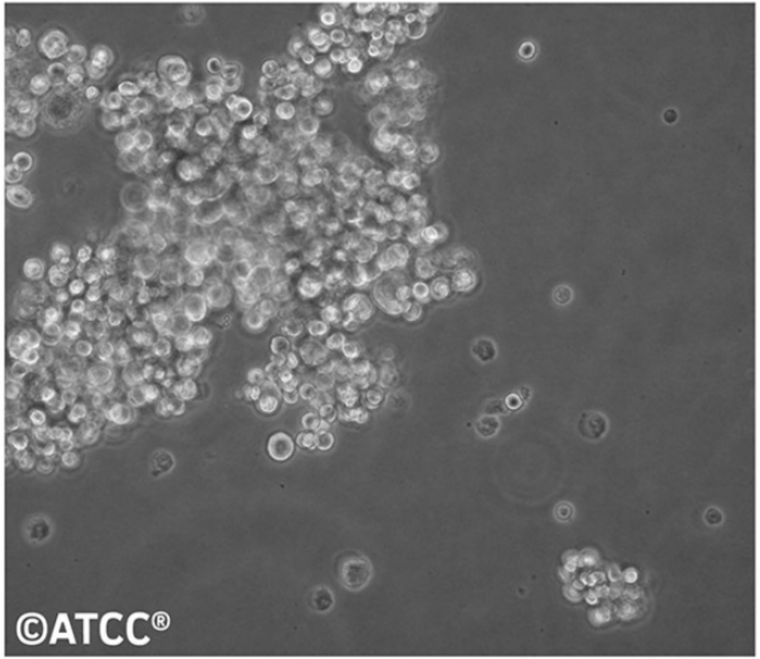

Low Density

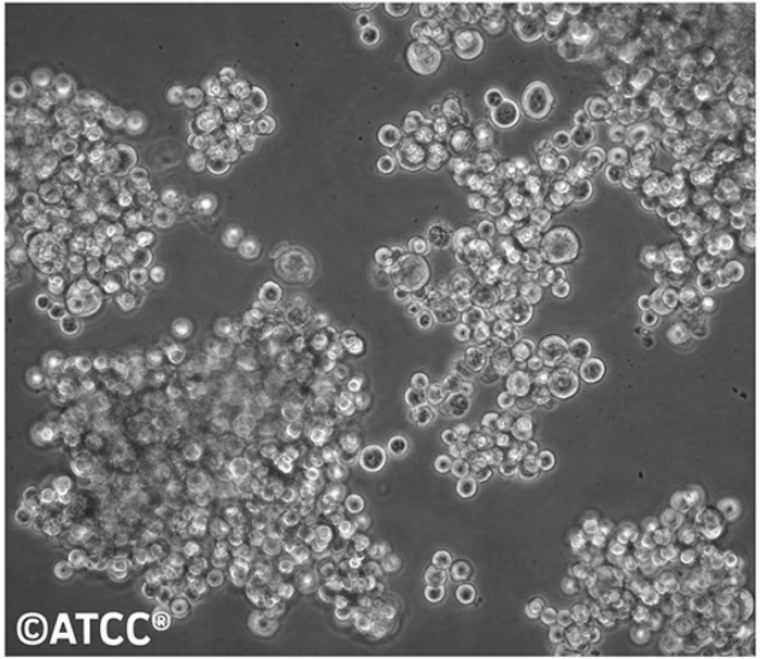

High Density

Supplementary Figure 13. Source: ATCC

OCUB-F

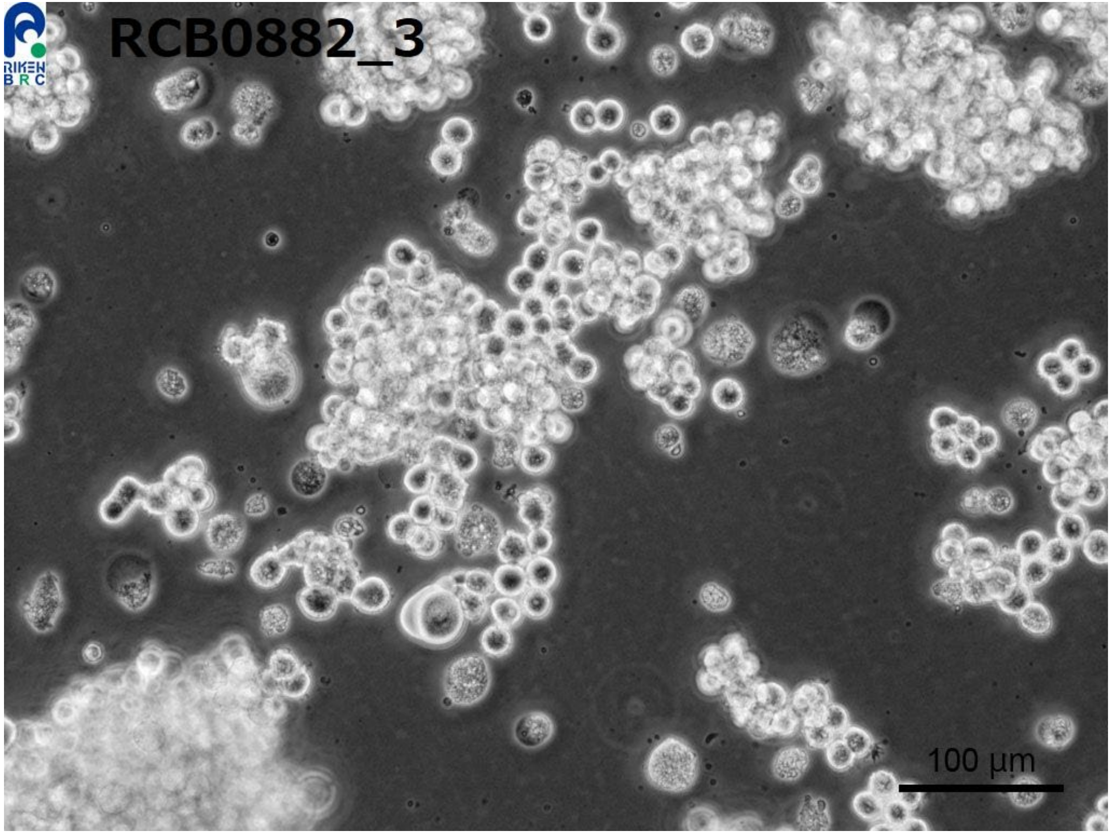

Supplementary Figure 14. Source: https://cellbank.brc.riken.jp/cell_bank/CellInfo/?cellNo=RCB0882

MDA-MB-468

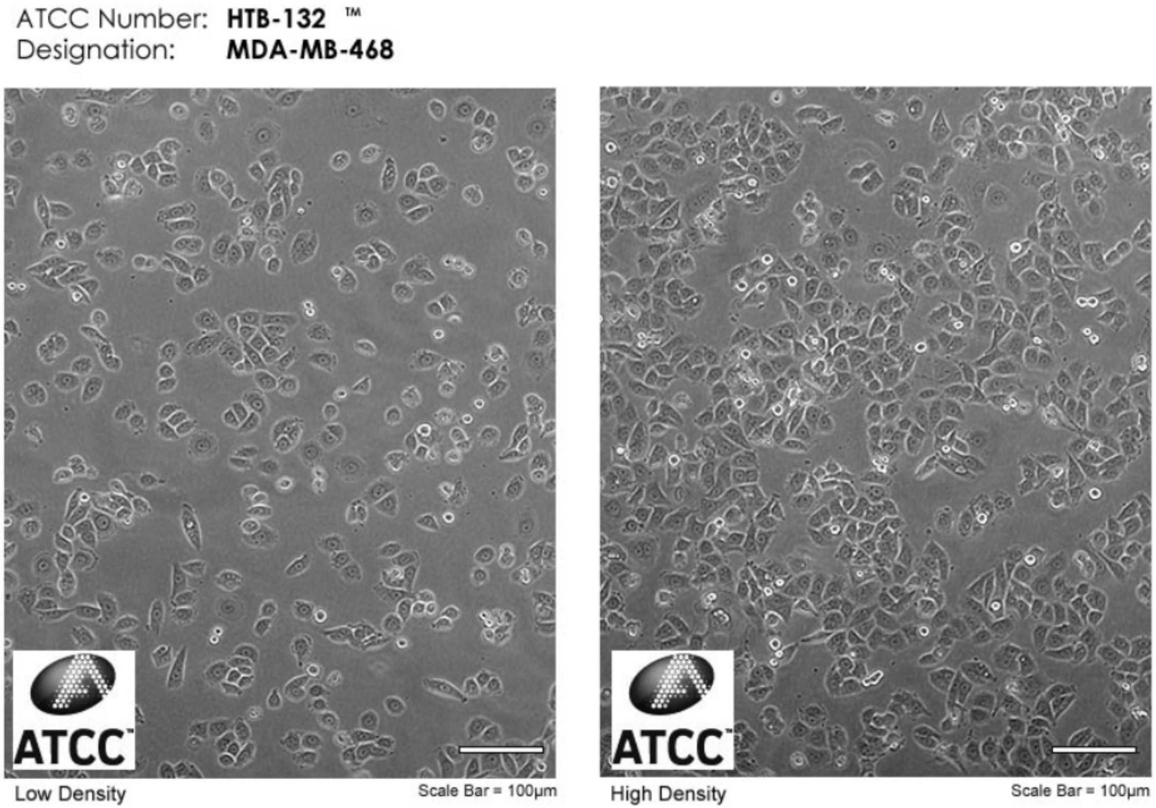

Supplementary Figure 15. Source: ATCC

CAL-120

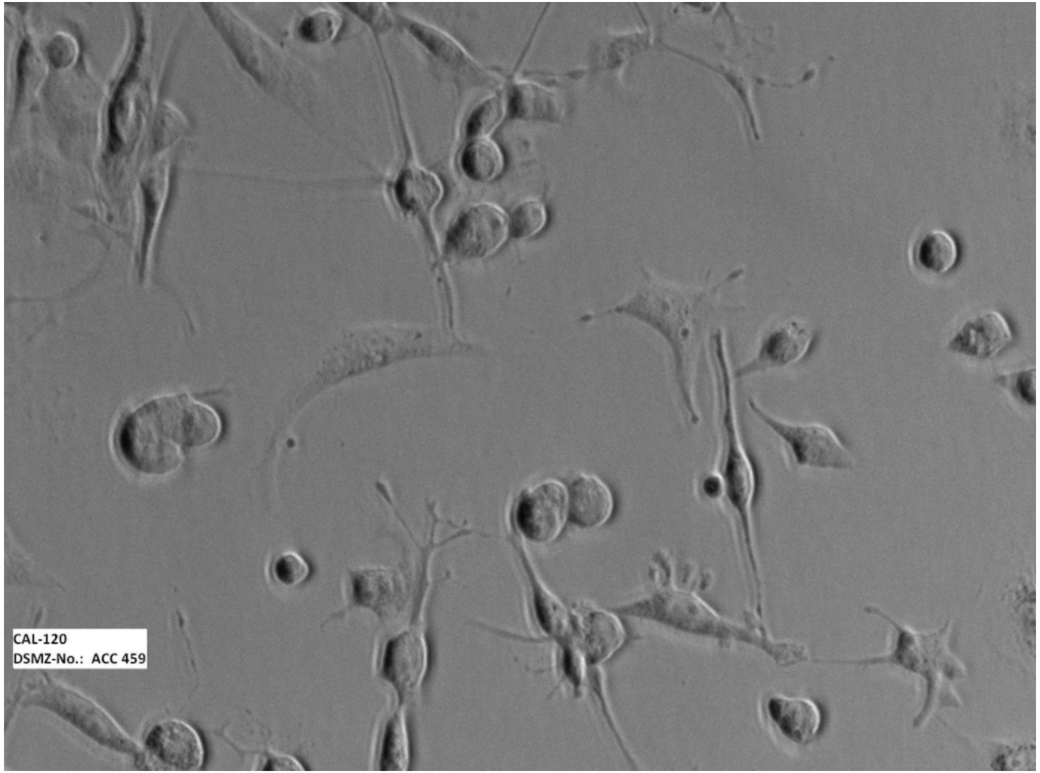

Supplementary Figure 16. Source: DSMZ

CAL-148

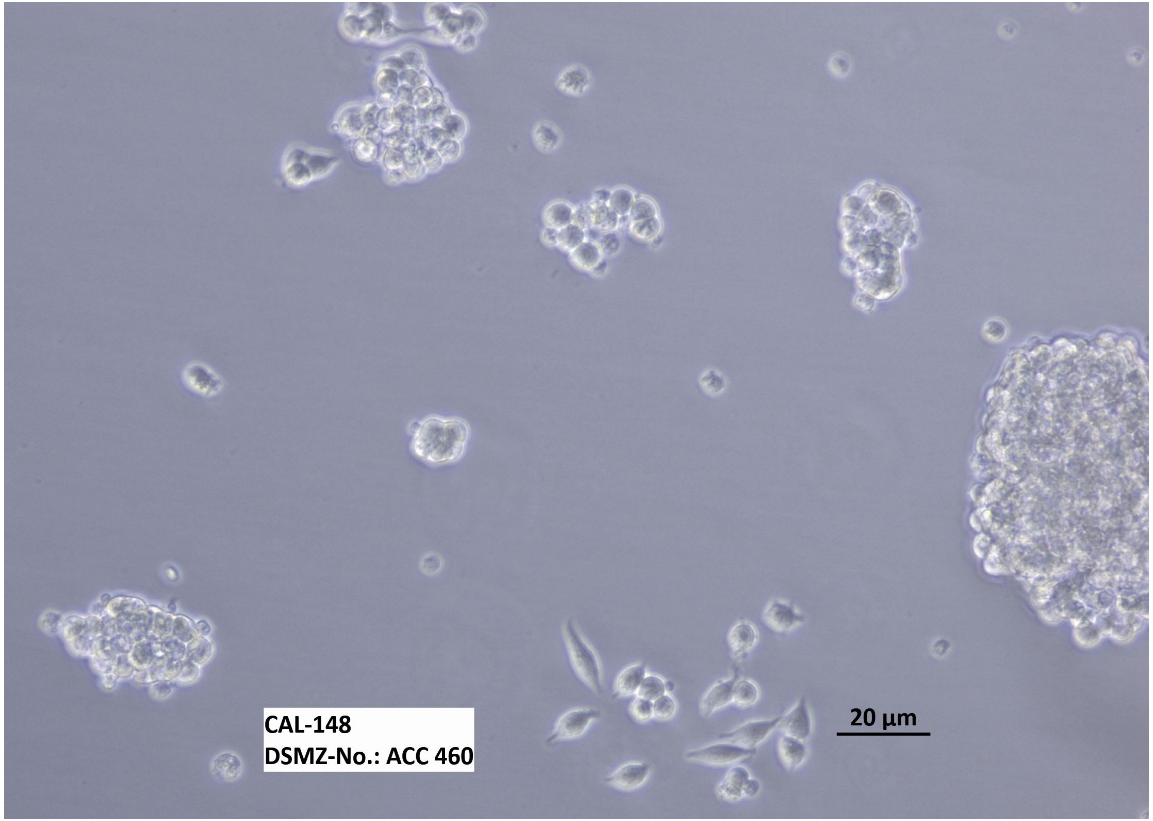

Supplementary Figure 17. Source: DSMZ

BT-549

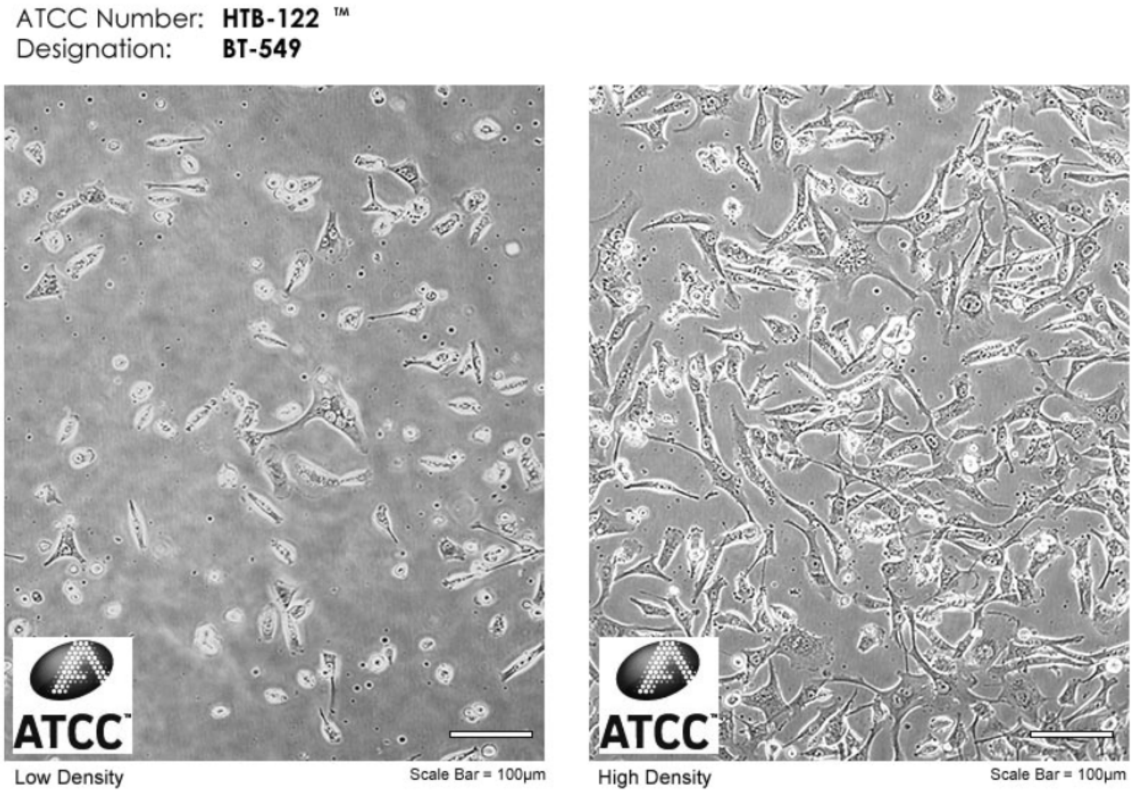

Supplementary Figure 18. Source: ATCC

SK-BR-3

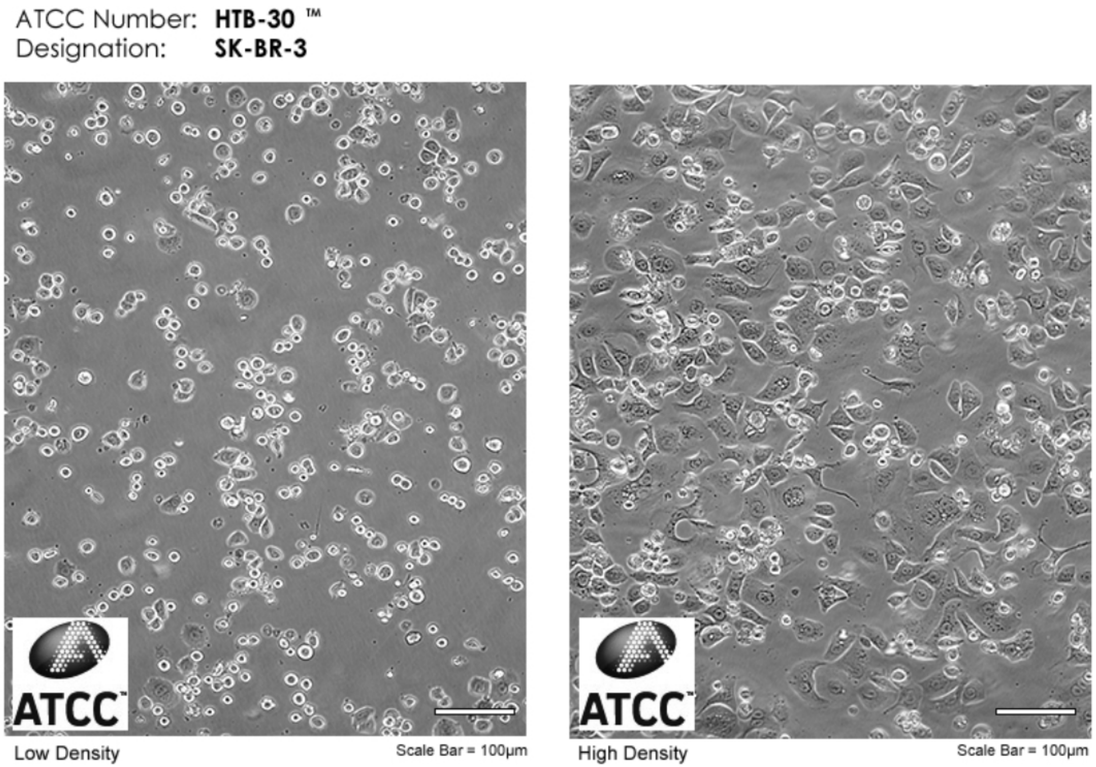

Supplementary Figure 19. Source: ATCC

SK-BR-5*

*No image found

Supplementary Figure 20. Source: ATCC
